# Supplementary figures and images for: EPOS trial: the effect of air filtration through a plasma chamber on the incidence of surgical site infection in orthopaedic surgery: a study protocol of a randomised, double-blind, placebo-controlled trial
Source: BMJ Open. 2022 Feb 3;12(2):e047500. doi: 10.1136/bmjopen-2020-047500 (PMC8814745; doi:10.1136/bmjopen-2020-047500)

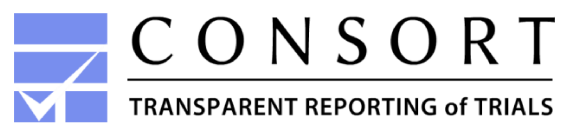

CONSORT Flow Diagram – Study plan for EPOS

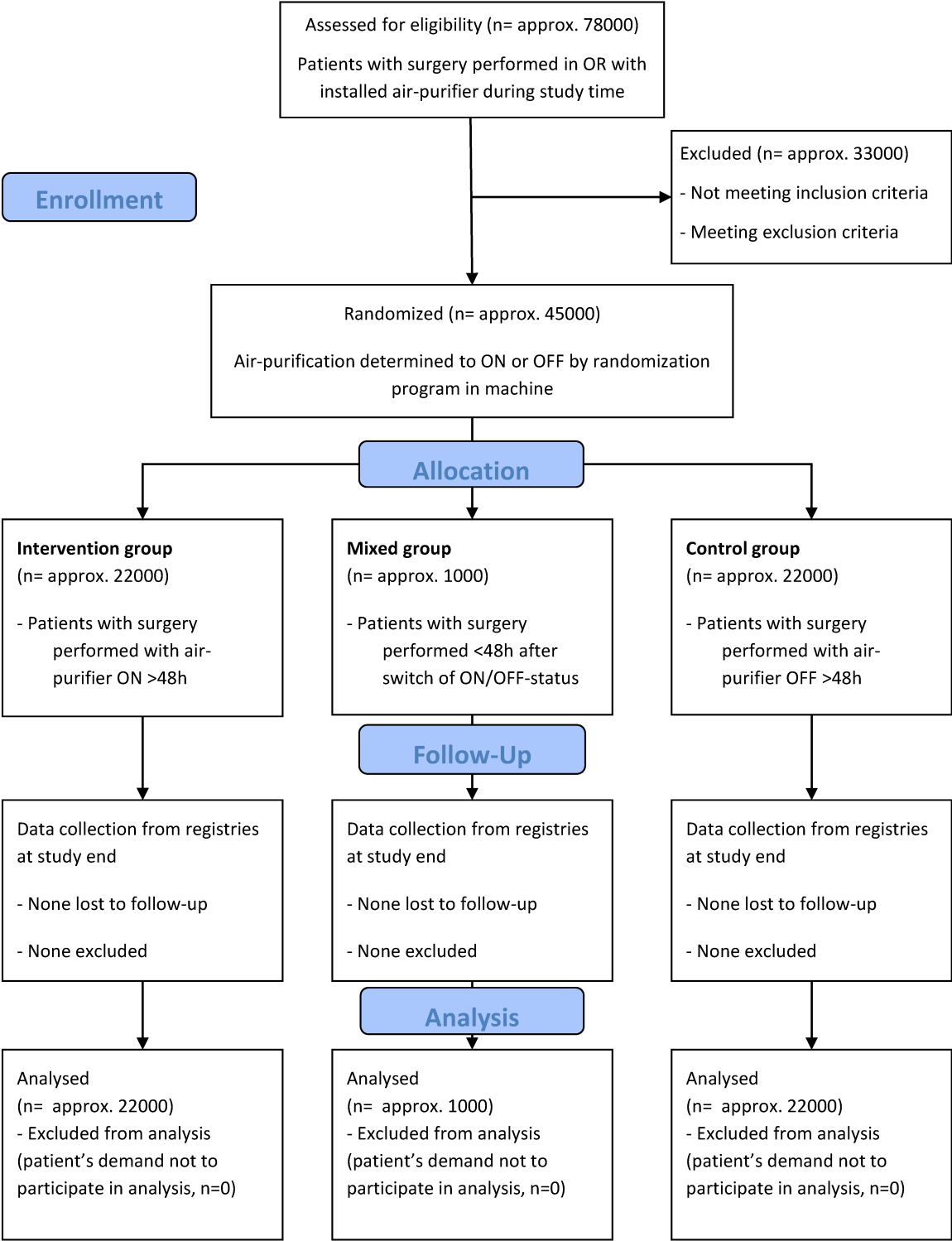

Supplement: Supplementary data [file bmjopen-2020-047500supp003.pdf]
