# Supplementary material for: EPOS trial: the effect of air filtration through a plasma chamber on the incidence of surgical site infection in orthopaedic surgery: a study protocol of a randomised, double-blind, placebo-controlled trial
Source: BMJ Open. 2022 Feb 3;12(2):e047500. doi: 10.1136/bmjopen-2020-047500 (PMC8814745; doi:10.1136/bmjopen-2020-047500)
Supplement: Supplementary data [file bmjopen-2020-047500supp001.pdf]

Appendix 1

1 R-script power analysis

```
# A certain number of infections in the dataset will not be due
# to the surgery that will be adding noise ot the calcualtion.
# We estimate this to 2% based on previous drug registry study experience
unrelated_rate <- 2/100
sig_lvl <- 0.05 # 0.05 with multiplicity for interim analysis of 0.005 added

##############################################
# Power calculation for all regular theaters #
##############################################
# The effect size in a regular operating theater
efx_size <- 0.4
# Base rate of SSI
inf.rate_base <- 2/100
# The SSI rate after the effect
inf.rate_efx <- inf.rate_base*(1-efx_size)
# The power is a combination of the unrelated rate + the rates of interest
power.prop.test(p1 = unrelated_rate + inf.rate_base,
                p2 = unrelated_rate + inf.rate_efx,
                sig.level = sig_lvl,
                power = 0.8)


##########################################
# Power calculation for a combination of #
# regular and ultra-clean theaters       #
##########################################
# The reduced effect in ultra-clean environments
efx_size_clean <- 0.25
# The expected infection rate in the ultraclean operating theaters
inf.rate_clean <- inf.rate_base * 0.8
# The proportion of ultra-clean operating theaters
prop_clean <- 0.8
# The SSI rate when combining the effect in regular and ultraclean operating theaters
inf.mix <- inf.rate_base * (1-prop_clean) +
  inf.rate_clean * prop_clean
# The reduced rate for above combination
inf.mix_efx <- inf.rate_base *
  (1-prop_clean) *
  (1-efx_size) +
  inf.rate_clean *
  prop_clean *
  (1-efx_size_clean)
# The power is a combination of the unrelated rate + the rates of interest
power.prop.test(p1 = unrelated_rate + inf.mix,
                p2 = unrelated_rate + inf.mix_efx,
                sig.level = sig_lvl,
                power = 0.8)
```
