# Supplementary material for: EPOS trial: the effect of air filtration through a plasma chamber on the incidence of surgical site infection in orthopaedic surgery: a study protocol of a randomised, double-blind, placebo-controlled trial
Source: BMJ Open. 2022 Feb 3;12(2):e047500. doi: 10.1136/bmjopen-2020-047500 (PMC8814745; doi:10.1136/bmjopen-2020-047500)
Supplement: Supplementary data [file bmjopen-2020-047500supp002.pdf]

Appendix 2

Informed consent material (freely translated from Swedish)

Since December 2016, there is an ongoing randomized trial at the XXXX Hospital, in which all patients that undergo orthopaedic surgery are included. The trial is performed to evaluate a new technique for air-purification in the operating rooms. We will perform follow-ups of all patients treated with surgery in those operating rooms, to check if the new air-purification has reduced the infection rate. Due to the large number of included patients in the study (approx. 45 000), the only data compiled for analysis will be the one already collected for mandatory national registries. If you had surgery performed at our department during this period of time, and wish more information regarding this study, or if you wish not to be included in the analysis part of the study, please contact any of the persons listed below:

Anders Persson	Max Gordon	Olof Sköldenberg
e-mail:	e-mail:	e-mail:
tel:	tel:	tel:
role:	role:	role:
