# Supplementary material for: EPOS trial: the effect of air filtration through a plasma chamber on the incidence of surgical site infection in orthopaedic surgery: a study protocol of a randomised, double-blind, placebo-controlled trial
Source: BMJ Open. 2022 Feb 3;12(2):e047500. doi: 10.1136/bmjopen-2020-047500 (PMC8814745; doi:10.1136/bmjopen-2020-047500)
Supplement: Author's manuscript [file bmjopen-2020-047500.draft_revisions.pdf]

BMJ Open

BMJ Open is committed to open peer review. As part of this commitment we make the peer review history of every article we publish publicly available.

When an article is published we post the peer reviewers' comments and the authors' responses online. We also post the versions of the paper that were used during peer review. These are the versions that the peer review comments apply to.

The versions of the paper that follow are the versions that were submitted during the peer review process. They are not the versions of record or the final published versions. They should not be cited or distributed as the published version of this manuscript.

BMJ Open is an open access journal and the full, final, typeset and author-corrected version of record of the manuscript is available on our site with no access controls, subscription charges or pay-per-view fees (http://bmjopen.bmj.com).

If you have any questions on BMJ Open's open peer review process please email info.bmjopen@bmj.com

BMJ Open

# Study protocol: The EPOS-trial - The effect of air-filtration through a plasma chamber on the incidence of surgical site infection in orthopaedic surgery. A randomized, double-blind, placebo-controlled trial

| | |
|---|---|
| Journal: | *BMJ Open* |
| Manuscript ID | bmjopen-2020-047500 |
| Article Type: | Protocol |
| Date Submitted by the Author: | 02-Dec-2020 |
| Complete List of Authors: | Persson, Anders; Karolinska Institutet Institutionen för kliniska vetenskaper Danderyds sjukhus,<br>Atroshi, Isam; Lund University, Department of Clinical Sciences; Hässleholm-Kristianstad Hospitals, Department of Orthopaedics<br>Tyszkiewicz, Thomas; Lund University, Department of Clinical Sciences; Hässleholm-Kristianstad Hospitals, Department of Orthopaedics<br>Hailer, Nils; Uppsala University, Department of Surgical Sciences; Uppsala University Hospital, Department of Orthopaedics<br>Lazarinis, Stergios; Uppsala University, Department of Surgical Sciences; Uppsala University Hospital, Department of Orthopaedics<br>Eisler, Thomas; Karolinska Institutet Institutionen för kliniska vetenskaper Danderyds sjukhus<br>Brismar, Harald; Karolinska Institute Department of Clinical Science Intervention and Technology; Karolinska Universitetsjukhuset i Huddinge, Department of Orthopaedics and Biotechnology<br>Mukka, Sebastian; Umea University, Department of Surgical and Perioperative Sciences; Umeå University Hospital<br>Kernell, Per-Juan; Löwenströmska Hospital, GHP Ortho Center Stockholm<br>Mohaddes, Maziar; Swedish Hip Arthroplasty Register; Institute of Clinical Sciences. Sahlgrenska Academy. University Of Gothenburg, Department of Orthopaedics<br>Sköldenberg, Olof; Karolinska Institutet Institutionen för kliniska vetenskaper Danderyds sjukhus<br>Gordon, Max; Karolinska Institutet Institutionen för kliniska vetenskaper Danderyds sjukhus |
| Keywords: | Orthopaedic & trauma surgery < SURGERY, Infection control < INFECTIOUS DISEASES, Hip < ORTHOPAEDIC & TRAUMA SURGERY, Adult orthopaedics < ORTHOPAEDIC & TRAUMA SURGERY, Knee < ORTHOPAEDIC & TRAUMA SURGERY |
| | |

SCHOLARONE™
Manuscripts

# Study protocol: The EPOS-trial - The effect of air-filtration through a plasma chamber on the incidence of surgical site infection in orthopaedic surgery. A randomized, double-blind, placebo-controlled trial

Anders Persson[1], Isam Atroshi[2], Thomas Tyszkiewicz[2], Nils Hailer[3], Stergios Lazarinis[3], Thomas Eisler[1], Harald Brismar[4], Sebastian Mukka[5], Per-Juan Kernell[6], Maziar Mohaddes[7], Olof Sköldenberg[1], Max Gordon[1]

1. Karolinska Institutet, Department of Clinical Sciences at Danderyd Hospital, Stockholm, Sweden
2. Lund University, Department of Clinical Sciences, Orthopaedics, Lund, and Department of Orthopaedics, Hässleholm-Kristianstad Hospitals, Kristianstad, Sweden
3. Uppsala University, Department of Surgical Sciences, Orthopaedics, Uppsala University Hospital, Uppsala, Sweden
4. Karolinska Institutet, Department of Clinical Science, Intervention and Technology, Department of Orthopaedics and Biotechnology, Karolinska University Hospital in Huddinge, Stockholm, Sweden
5. Umeå University, Department of Surgical and Perioperative Sciences, Umeå University Hospital, Umeå, Sweden
6. GHP Ortho Center Stockholm, Löwenströmska Hospital
7. Department of Orthopaedics, Institute of Clinical Sciences, Sahlgrenska Academy, Gothenburg University, Gothenburg, Sweden, and The Swedish Arthroplasty Register, Gothenburg, Sweden

Correspondance: Anders Persson, M.D., PhD student, Department of Clinical Sciences, Dep. of Orthopaedics, Karolinska Institutet at Danderyd Hospital, S-18288 Danderyd, Sweden, tel: +46 8 12355000

E-mail: anders.persson@sll.se

**Key words:** surgical site infection, prevention, RCT, air-filtration, operating room, environment

**Word count:** 2961

For peer review only

# Abstract

### *Introduction*

There is controversy regarding the importance of air-transmitted infections for surgical site infections (SSI's) after orthopaedic surgery. Research has been hindered by both the inability in blinding the exposure, and by the need for recruiting large enough cohorts. The aim of this study is to investigate whether using a new form of air-purifier using plasma-air-purification (PAP) in operating rooms (OR's) lower the SSI-rate, or not.

### *Methods and analysis*

Multicenter, double-blind, cluster-randomized, placebo-controlled trial conducted at seven hospitals 2017-2022. All patients that undergo orthopaedic surgery for minimum 30 minutes are included. Intervention group: patients operated in OR with PAP-devices turned ON. Control group: patients operated in OR with PAP-devices turned OFF. Randomization: Each OR will be randomized in periods of 4, 6, or 8 weeks to either have the devices on or off. Primary outcome: Any SSI postoperatively defined as a composite endpoint of any of the following: use of isoxazolylpenicillin, clindamycin or rifampicin for 2 days or more, ICD codes or Nordic Medico-Statistical Committee (NOMESCO) codes indicating postoperative infection. In a second step, we will perform a chart review on those patients with positive indicators of SSI to further validate the outcome. Secondary outcomes are described in the methods' section. Power: We assume an SSI rate of 2%, an SSI reduction rate of 25%, and a majority of ultra-clean OR's, which gives us approx. 45 000 patients to attain a power of 80% and significance level of 0.05.

### *Ethics and dissemination*

The study is approved by the Swedish Ethical Review Authority. The interim analysis results from the study will be presented only to the researchers involved unless the study thereafter is interrupted for whatever reason. Publication in a medical journal will be presented after inclusion of the last patient.

### *Registration details*

The EPOS trial is registered at ClinicalTrials.gov with identifier NCT02695368.




For peer review only - http://bmjopen.bmj.com/site/about/guidelines.xhtml

## Strengths and limitations of this study

- ✓ Multicenter, placebo-controlled trial with approximately 45 000 study subjects
- ✓ Double-blinded
- ✓ Cluster-randomization design that switches within each operating room minimizes risk of allocation bias
- ✓ Unusual outcome requires large number of study subjects, which makes the project resource intensive

For peer review only

## Introduction

Despite surgery in clean OR's, surface sterilization, and antibiotics, SSI after orthopaedic surgery have an overall estimated incidence of 1-4% (1-3). This feared complication is associated with long-term antibiotics, repeated surgeries, prolonged hospital stays, economic burden, and a poorer end result for individual patients (4, 5). Prevention of SSI's is therefore of paramount importance.

Air flow within the OR can spread airborne particles, posing a potential risk for postoperative infection. These airborne particles include dust, textile fibres, skin scales, and respiratory aerosols, loaded with viable microorganisms (including *Staphylococcus aureus*) having been released mainly from the surgical team members and patient into the surrounding air of the OR. These particles have been shown to settle onto surfaces including the surgical wound and instruments (6). Thus, air-transmitted infections is one of the main reasons for SSI (6), making this an interesting intervention target.

Ever since the ground-breaking study by Charnley in 1969 (7), which showed that cleaner air in the OR drastically improved infection rates after total hip arthroplasty, vast efforts have been made to address this issue. Lidwell carried on his work, showing that ultra-clean air (i.e. <10 colony-forming units (CFU)/$m^3$) in combination with body-exhaust suits and prophylactic antibiotics further improved infection rates (8-10). Based on their work, in combination with several reports showing that laminar air flow (LAF) reduces bacterial contamination in the OR air (11-13), many OR's are today equipped with LAF ventilation. Unfortunately though, when evaluated in large cohort studies, systematic reviews and meta-analyses, LAF systems in modern state-of-the-art OR's have so far failed to prove efficient in preventing infections, compared to conventional ventilation (14-19).

The plasma air-purification (PAP) system used in our current study is an air purifier that sterilizes the air particles through a plasma chamber. Air in the OR is pumped through the chamber, and by using a small current it transforms the air in the vicinity of the electrode into plasma, which eradicates any bacteria that pass through. The small size of the machine allows it to fit into any operating theatre without interfering with existing equipment (20).

Similar systems exists that use ultraviolet light to sterilize air particles and reduce the rate of hospital acquired infections (21, 22). There are though few peer-reviewed articles regarding air-purification. While we have not found any randomized clinical trials, there are randomised field trials, outside hospital settings, that have shown positive effects in vivo. In a blinded randomized field trial on healthy volunteers using air purifiers, a significant reduction in air-particles were seen and this also led to a reduction of stress hormone for the participants. (23)

In hospital and OR settings the PAP technology alone significantly reduces the number of colony-forming units (CFUs) of staphylococci (the most common infecting microorganism in SSI) from 49-97% (24). In non-randomized studies it has been shown to reduce respiratory infections, personnel sick-leave, and severe infectious outbreaks (22, 24). These effects have though not been validated in randomized clinical trials in operating room settings. However, this is also true for all methods of reducing air-borne pathogens in operating rooms

For peer review only


For peer review only - http://bmjopen.bmj.com/site/about/guidelines.xhtml

that currently are used, such as surgery in LAF OR's. The need for Level 1 evidence in this field of medicine is urgent.

The EPOS-trial is therefore designed as a multicentre, double-blinded, cluster-randomized, placebo-controlled trial.

The aim of this study is to investigate whether using PAP devices that clean the air from contaminating particles in OR's lower the rate of SSI, or not. The primary endpoint will be SSI rate, through a proxy variable described in the methods section, and we hypothesize that PAP can reduce the incidence of SSI in orthopaedic surgery by 25%. Secondary aims include investigating the number of prescribed antibiotics as well as the number of needed readmissions and length-of-stay for SSI.

For peer review only

## Methods: Participants, interventions and outcomes

### *Study setting*

The study is being conducted at seven major hospitals in Sweden. Inclusion will take place between 2017 and 2022. The randomized clinical trial setting has been chosen to control for the huge number of possible confounders influencing the outcome. The study protocol has been written according to the SPIRIT (Standard Protocol Items for Randomized Trials) statement.

### *Eligibility criteria*

We will **include** (1) all patients that undergo surgery for 30 minutes or longer at each centre during the study period. We assume that surgeries lasting less than 30 minutes are less susceptible to SSI's. Including those in this study would therefore result in a larger cohort. We will **exclude** surgeries on 1) already infected surgical sites, defined as: ICD- or NOMESCO codes indicating infection (same as those used for the primary outcome, see below), open fractures, traumatic wounds, vacuum assisted wound therapy, 2) patients that have withdrawn antibiotics 2 weeks or less prior to surgery, since these are either more probable to already be infected, or less probable to acquire an SSI due to antibiotic protection. If patients have multiple surgeries during the study period, only the first operation will be included.

### *Intervention*

At each hospital, all OR's that perform surgery on orthopaedic patients will be equipped with three (3) PAP-systems each. The groups are defined as: **Intervention group:** those operated where the PAP device has been turned on for at least 2 days prior to index surgery. **Control group:** those where the PAP device has been turned off for at least 2 days prior to index surgery. **Mixed group:** those receiving surgeries in OR's within 2 days after the PAP device switches status. We will also in the analysis sub-group the study subjects according to measurements prior to study start into: ***Regular*** operating rooms (≥10 CFU/ $m^3$) and ***Ultra-clean*** operating rooms (<10 CFU/$m^3$).

PAP device status and function will be monitored continuously during the inclusion period through standardized manual controls every 3 months, and also at the end of the inclusion period by validating PAP device status retrospectively through memory card recordings in each device.

### *Outcomes*

The **primary outcome** is any indication of SSI within 12 weeks postoperatively, defined as a composite endpoint of any of the following:

1. Withdrawal or other documented use of antibiotics corresponding to 2 days or more after surgery targeting Staphylococcus aureus
2. ICD code indicating postoperative infection (at date of readmission)
3. NOMESCO code indicating postoperative infection


For peer review only - http://bmjopen.bmj.com/site/about/guidelines.xhtml

In a second step, we will perform a chart review on those patients with positive indicators of SSI to further validate the outcome. We will use the internationally accepted CDC definition of SSI when finally establishing that a SSI has occurred (Table 1) (25).

The **effect size** of the primary endpoint is the relative risk of contracting SSI for the intervention group versus the control group and will be calculated by dividing the probability of contracting a SSI in the intervention group by the control group = $\frac{SSI\ rate\ intervention\ group}{SSI\ rate\ control\ group}$.
The **effect size** will also be presented as an absolute risk difference = $SSI\ rate\ intervention\ group - SSI\ rate\ control\ group$.

The primary outcome is a **surrogate variable** for SSI as it due to the large sample size is practically impossible to have all patients come back for outpatient visits and be visually inspected. The Swedish health-care registers, especially the Prescribed Drug Register and the Patient Register will make sure that we get an almost complete (>99%) coverage of all relevant SSI's. In a second step, a chart review from all hospital charts will be performed on all individual patients that have codes or prescriptions indicating SSI.

The **secondary outcomes** are:

a) Withdrawal or other documented use of any antibiotics for 2 days or more after surgery during the first 30 postoperative days
b) Number of days with antibiotics during the first 30 days
c) Same as a) & b) but up to 90 days after surgery
d) Death during the first 2 postoperative years

These analyses will also be performed with and without adjustment for pre-operative antibiotic use 6 months prior to the surgery.

### *Sample size*

The re-operation rate in Sweden due to infections within the first 2 years after surgery is 1.3% (3) for primary total hip replacements (THR's). This does not include THR's in fracture patients and other types of surgeries that are more susceptible to infections. We therefore assume that the SSI rate in our study population is 2% (1-3). Similarly, we know from other data that about 0.7% withdraw the antibiotics associated with the primary outcome within a 3 months' period prior to surgery. To account for infections unrelated to surgery we assume that the infection noise rate, i.e. non-surgical site infections, is less than 2%.
***Multicentre power with ultra-clean air:*** Hospitals with OR's with ultra-clean air may be less susceptible to the effect with an already lower infection rate. Pre-study CFU measurements suggest that approximately 80% of the included OR's are ultra-clean. If we assume that the infection rate is 2% or less, and that the effect size is reduced to 25% in an ultra-clean environment, we will need to recruit 22,630 patients in each group, i.e. approximately 45,000 patients to attain a power of 80% with a significance level of 0.05. This will be our target population. We expect a very low drop-out rate/missing data in the study, estimated to be <1%. At the interim analysis the p-value will be set 10 times lower; at 0.005; i.e. if a statistically significant result between the groups is observed at 18 months, the study will be stopped. See Figure 1 and Appendix 1 for details in R and explanation of the script for the sample size analysis.


For peer review only - http://bmjopen.bmj.com/site/about/guidelines.xhtml

### *Recruitment*

We estimate that with only one center, such as Danderyd Hospital, recruitment would take >5 years. The hospital operates about 4,500 patients each year and as it is unlikely that more than 500 of these will be excluded. We therefore anticipate recruiting 4,000 patients/year. Performing this study in a multi-center setting is therefore crucial. In Table 2 the participating centers are presented, and their estimated proportion of patients included.

### *Patient and Public Involvement*

Representatives of the Swedish Osteoporosis Society (www.osteoporos.org) and the Swedish Rheumatic Society (www.reumatiker.se) are members of the study steering committee, and participate mainly in discussions regarding plans for reporting and publishing the results of the study.

For peer review only



For peer review only - http://bmjopen.bmj.com/site/about/guidelines.xhtml

## Methods: Assignment of interventions

### *Allocation*

The three PAP devices in each OR will synchronically be randomized in periods of 4, 6, or 8 weeks to either have the system “on” or “off”. The switch will always occur midnight Friday in order to limit the patients exposed to partial effect during the first two days after switching status. The system can be programmed to be active (i.e. plasma chamber eradicating bacteria) at any given timeframe. The manufacturer of the PAP devices will prepare the randomization allocation and automatic execution of it. The randomization sequence is at minimum 8 years long and will be submitted to a third, independent party, responsible for keeping the allocation secret until interim analyses or study end. At the interim analyses only allocations up to that date will be released.

### *Blinding*

The on/off only refers to the plasma chamber responsible for the antimicrobial effect. As the machine retains the air flow it will be impossible for staff, surgeons and patients to determine the status of the machine from the outside. The device will also automatically switch status where the true status is concealed for all study participants including other hospital personnel until the end of the study.

For peer review only

# Methods: Data collection, management, and analysis

### *Data collection methods*

For the primary endpoint the following codes will be used to detect if individual patients have contracted an SSI following surgery. If any of these codes indicate SSI, a chart review will be performed in a second step to verify the outcome:

1. From The Swedish Prescribed Drug Register: withdrawal of antibiotics targeting Staphylococcus aureus corresponding to a minimum amount of 2 defined daily dosages (an estimate provided by the registry for the expected daily dosage). The date of withdrawal will serve as an indicator of treatment start unless inpatient data is available with more granular information. The drug ATC codes considered to target relevant bacteria are:
    a. J01CF05 (isoxazolylpenicillin)
    b. J01FF01 (clindamycin)
    c. J04AB02 (rifampicin)
2. From The National Patient Registry: ICD trigger codes indicating postoperative infections:
    a. T793 – Post-traumatic wound infection, not elsewhere classified
    b. T814 – Infection following a procedure, not elsewhere classified
    c. T84[5-7] – Infection and inflammatory reaction due to internal joint prosthesis, internal fixation device, or other internal orthopaedic prosthetic devices, implants and grafts
    d. T874 – Infection of amputation stump
    e. B9[5,6,8] – Bacterial specification
    f. L0[2-4] – Cutaneous abscess, furuncle and carbuncle; cellulitis
    g. A[24]6 – Erysipeloid, erysipelas
3. From The National Patient Registry: NOMESCO trigger codes indicating postoperative infections:
    a. Incision abscess: TN[A-H]05
    b. Surgeries due infections: N[A-H]S[0-4,9]9
    c. Extremities wound revision: Q[CD]B05
    d. Re-operation for infection: N[A-H]W69
    e. Vacuum treatment: DQ023

Both local and national registry data will be used according to availability. For the admission episodes with the code indicators the admission date is the index date, i.e. if an admission occurs after 91 days with a trigger code it will not be considered an indicator of a postoperative infection.

In-hospital information systems will supply information on:

a) Patient ID
b) Date(s) of surgery
c) Surgery associated codes including operated side
d) Operating room
e) In-hospital antibiotics


For peer review only - http://bmjopen.bmj.com/site/about/guidelines.xhtml

Both the surgical and medical data records will be retrieved depending on availability. Only centres that can provide the above data will be allowed to participate.

The Swedish National Patient Register includes all in-patient care and outpatient visits in Sweden with discharge codes according to ICD-10, NOMESCO codes and admission/discharge dates (26).

The Swedish Prescribed Drug Register (PDR) includes any withdrawn prescriptions. Prescriptions that are never withdrawn by patients and drugs bought over the counter without prescriptions are not included. The data fields used were the drug ATC-code, number of pills, and prescription text (27).

### *Data management*

The study data will be securely managed and stored encrypted at a computer within Karolinska Institutet at Danderyd Hospital. No other than the authors stated above will gain access to raw data.

### *Statistical methods*

The primary outcome is a binary variable where there are three groups. We will use logistic regression where the reference group is the placebo group, and the significance is related to the intervention group. The estimate for the mixed group is only for relating dose-effect, i.e. the group will not be pooled with either the placebo or the intervention group. Due to the randomization we do not intend to have any other covariates as confounders in the model. Similar methodology will be applied to the antibiotic's binary outcome. The number of days with antibiotics will be modelled using a linear regression with the similar interpretation to above regarding predictors. Mortality will be modelled using a cox proportional hazards model with time since surgery calculated as time to death, migration or 2 years. The cox model will not contain any covariates due to the randomized study nature. The analysis will be performed by an epidemiologist/statistician in our team (MG) and will be performed as a per-protocol analysis.

We will handle confounding by including only the patient's 1st surgical procedure. The randomization process will handle other confounding such as confounding by selection. Procedural confounding will be handled by the external part who has done the randomization procedure for each PAP device. Regarding dropouts the health-care registers and chart review done in the study will ensure a very low (<1%) drop-out rate. Individual patients can also request that they are excluded from the registers and thus from the study, but by experience, this is very rare and will also not affect our ability to analyse our primary and secondary endpoints.



For peer review only - http://bmjopen.bmj.com/site/about/guidelines.xhtml

# Methods: Monitoring

### *Data monitoring*

At 12 months after study start (2018-04-17) an interim analysis will be performed and the recruitment rate from each center will be evaluated. The study recruitment will end once we have reached a minimum of 45,000 patients. During the 2nd half of 2017/early 2018, the data quality of each recruiting center will be evaluated by extracting data from each center's hospital information center.

For peer review only


For peer review only - http://bmjopen.bmj.com/site/about/guidelines.xhtml

## Ethics and dissemination

The study is being conducted in accordance with the ethical principles of the Helsinki Declaration, and is approved by the Swedish Ethical Review Authority (2015/1139-31/4).

The interim analysis results from the study will be presented only to the researchers involved unless the study thereafter is interrupted due to significant difference in infection rate between the two groups. Publication in a medical journal will be presented after inclusion of the last patient.

Study participant information will be published on the hospital web site (see Appendix 2). No personal consent forms will be collected

The EPOS trial is registered at ClinicalTrials.gov with identifier NCT02695368.

## Author contributions

AP operates the trial, and led the writing of this manuscript, with contributions from the rest of the authors. TE, MM, HB, NH, SL, TT, IA, SM and PK operate the inclusion centers. MG and OS designed the original study and developed the protocol. MG is the responsible statistician and supervises the study. All authors contributed to the editing and redrafting of the manuscript.

## Funding statement

8.8 million SEK from the Swedish Research Council has been received for 2017-2020 (grant number 2017-00198). 1.4 million SEK from ALF (Stockholm County and Karolinska Institute, application number 20160251, record number LS 2015-1198) has been received for 2017-2018. We have a discounted price for the rental of the machines but have chosen not to apply for funding from the manufacturers of the plasma-air-purifier equipment to ensure that the study is independent.

## Competing interests statement

None.

For peer review only


For peer review only - http://bmjopen.bmj.com/site/about/guidelines.xhtml

## References

1. Reilly J, Allardice G, Bruce J, Hill R, McCoubrey J. Procedure-specific surgical site infection rates and postdischarge surveillance in Scotland. Infection control and hospital epidemiology. 2006;27(12):1318-23.
2. Pull ter Gunne AF, Cohen DB. Incidence, prevalence, and analysis of risk factors for surgical site infection following adult spinal surgery. Spine. 2009;34(13):1422-8.
3. Lindgren JV, Gordon M, Wretenberg P, Kärrholm J, Garellick G. Validation of reoperations due to infection in the Swedish Hip Arthroplasty Register. BMC musculoskeletal disorders. 2014;15:384.
4. de Lissovoy G, Fraeman K, Hutchins V, Murphy D, Song D, Vaughn BB. Surgical site infection: incidence and impact on hospital utilization and treatment costs. Am J Infect Control. 2009;37(5):387-97.
5. Bozic KJ, Katz P, Cisternas M, Ono L, Ries MD, Showstack J. Hospital resource utilization for primary and revision total hip arthroplasty. J Bone Joint Surg Am. 2005;87(3):570-6.
6. Fernstrom A, Goldblatt M. Aerobiology and its role in the transmission of infectious diseases. Journal of pathogens. 2013;2013:493960.
7. Charnley J, Eftekhar N. Postoperative infection in total prosthetic replacement arthroplasty of the hip-joint. With special reference to the bacterial content of the air of the operating room. Br J Surg. 1969;56(9):641-9.
8. Lidwell OM, Elson RA, Lowbury EJ, Whyte W, Blowers R, Stanley SJ, et al. Ultraclean air and antibiotics for prevention of postoperative infection. A multicenter study of 8,052 joint replacement operations. Acta orthopaedica Scandinavica. 1987;58(1):4-13.
9. Lidwell OM, Lowbury EJ, Whyte W, Blowers R, Stanley SJ, Lowe D. Airborne contamination of wounds in joint replacement operations: the relationship to sepsis rates. J Hosp Infect. 1983;4(2):111-31.
10. Lidwell OM, Lowbury EJ, Whyte W, Blowers R, Stanley SJ, Lowe D. Effect of ultraclean air in operating rooms on deep sepsis in the joint after total hip or knee replacement: a randomised study. British medical journal (Clinical research ed). 1982;285(6334):10-4.
11. Hirsch T, Hubert H, Fischer S, Lahmer A, Lehnhardt M, Steinau HU, et al. Bacterial burden in the operating room: impact of airflow systems. Am J Infect Control. 2012;40(7):e228-32.
12. Diab-Elschahawi M, Berger J, Blacky A, Kimberger O, Oguz R, Kuelpmann R, et al. Impact of different-sized laminar air flow versus no laminar air flow on bacterial counts in the operating room during orthopedic surgery. Am J Infect Control. 2011;39(7):e25-9.
13. Romano F, Milani S, Ricci R, Joppolo CM. Operating Theatre Ventilation Systems and Their Performance in Contamination Control: "At Rest" and "In Operation" Particle and Microbial Measurements Made in an Italian Large and Multi-Year Inspection Campaign. Int J Environ Res Public Health. 2020;17(19).
14. WHO. Global Guidelines for the Prevention of Surgical Site Infection. WHO Guidelines Approved by the Guidelines Review Committee. Geneva2016.
15. Breier AC, Brandt C, Sohr D, Geffers C, Gastmeier P. Laminar airflow ceiling size: no impact on infection rates following hip and knee prosthesis. Infection control and hospital epidemiology. 2011;32(11):1097-102.
16. Gastmeier P, Breier AC, Brandt C. Influence of laminar airflow on prosthetic joint infections: a systematic review. J Hosp Infect. 2012;81(2):73-8.
17. Bischoff P, Kubilay NZ, Allegranzi B, Egger M, Gastmeier P. Effect of laminar airflow ventilation on surgical site infections: a systematic review and meta-analysis. Lancet Infect Dis. 2017;17(5):553-61.
18. McHugh SM, Hill AD, Humphreys H. Laminar airflow and the prevention of surgical site infection. More harm than good? Surgeon. 2015;13(1):52-8.

For peer review only

19. Pasquarella C, Barchitta M, D'Alessandro D, Cristina ML, Mura I, Nobile M, et al. Heating, ventilation and air conditioning (HVAC) system, microbial air contamination and surgical site infection in hip and knee arthroplasties: the GISIO-SItI Ischia study. Ann Ig. 2018;30(5 Supple 2):22-35.
20. Novaerus Technology 2020 [Available from: https://cmsifyassets-1290.kxcdn.com/nov-production/uploads/asset/1741/attachment/NV_NanoStrike_Brochure_HIRES.pdf.
21. Anis HK, Curtis GL, Klika AK, Piuzzi NS, Otiso J, Richter SS, et al. In-Room Ultraviolet Air Filtration Units Reduce Airborne Particles During Total Joint Arthroplasty. J Orthop Res. 2020;38(2):431-7.
22. Kane D. Study shows reduced HAIs, airborne pathogens with in-room active UV-C system http://www.mcknights.com/marketplace/study-shows-reduced-hais-airborne-pathogens-with-in-room-active-uv-c-system/article/573958/2017 [
23. Li H, Cai J, Chen R, Zhao Z, Ying Z, Wang L, et al. Particulate Matter Exposure and Stress Hormone Levels: A Randomized, Double-Blind, Crossover Trial of Air Purification. Circulation. 2017;136(7):618-27.
24. James E. Independent validation report for Novareus air treatment systems 2014 [Available from: http://sandbox.novaerus.com/wp-content/uploads/2014/03/Independent-Validation-Report-for-Novaerus-NHS.pdf.
25. Horan TC, Gaynes RP, Martone WJ, Jarvis WR, Emori TG. CDC definitions of nosocomial surgical site infections, 1992: a modification of CDC definitions of surgical wound infections. Infection control and hospital epidemiology. 1992;13(10):606-8.
26. The National Patient Register [Internet]. 2019. Available from: https://www.socialstyrelsen.se/en/statistics-and-data/registers/register-information/the-national-patient-register/.
27. Prescribed Drug Register [Internet]. 2019. Available from: https://www.socialstyrelsen.se/statistik-och-data/register/alla-register/lakemedelsregistret/.



For peer review only - http://bmjopen.bmj.com/site/about/guidelines.xhtml

# Figures

1. Sample size analysis

For peer review only


For peer review only - http://bmjopen.bmj.com/site/about/guidelines.xhtml

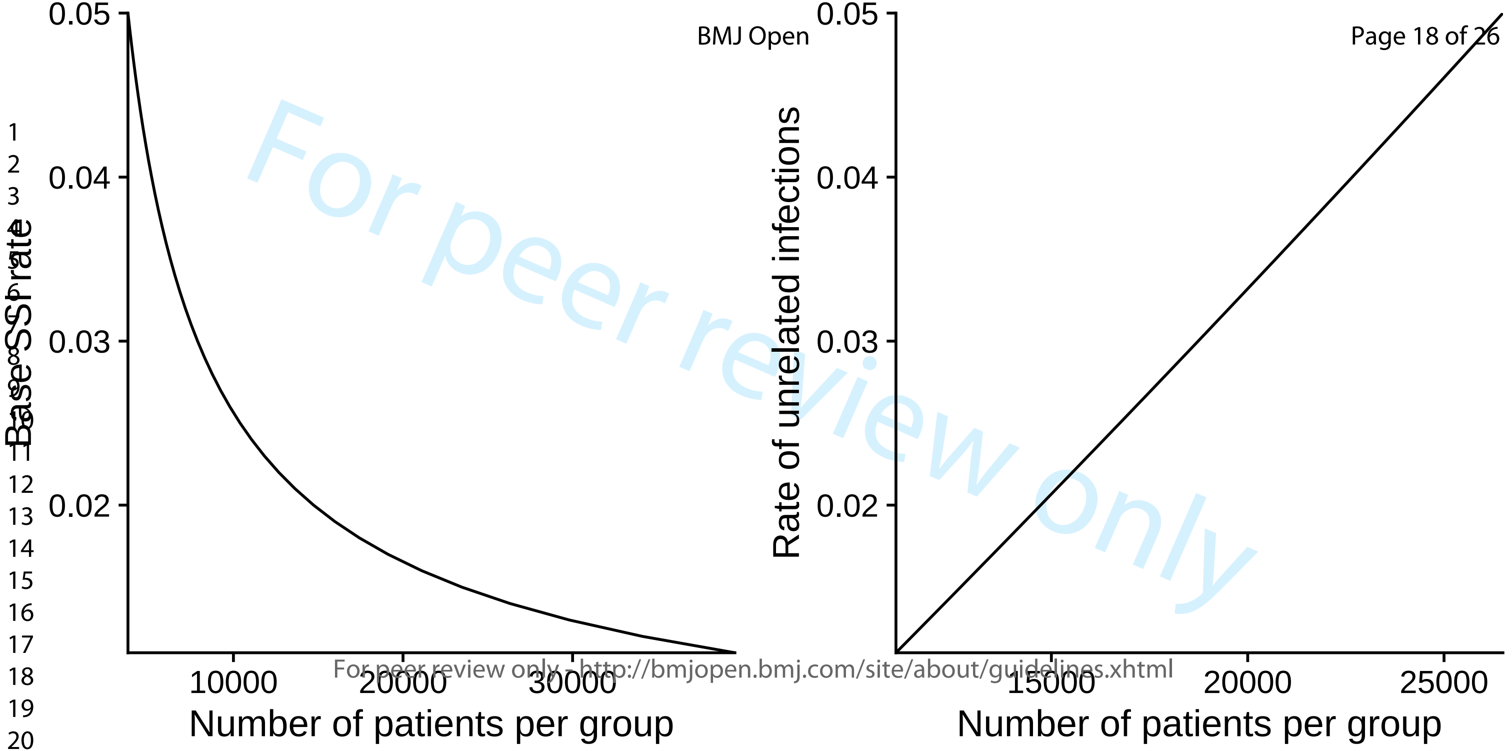


For peer review only

For peer review only - http://bmjopen.bmj.com/site/about/guidelines.xhtml

**Table 1: CDC criteria for surgical site infections**

**Superficial Incisional Surgical Site Infection**

Infection within 30 days after the operation and only involves skin and subcutaneous tissue of the incision and at least one of the following:

1. Purulent drainage with or without laboratory confirmation, from the superficial incision.
2. Organisms isolated from an aseptically obtained culture of fluid or tissue from the superficial incision.
3. At least one of the following signs or symptoms of infection: pain or tenderness, localised swelling, redness, or heat and superficial incision is deliberately opened by surgeon, unless incision is culture negative.
4. Diagnosis of superficial incisional SSI made by a surgeon or attending physician.

**Deep Incisional Surgical Site Infection**

Infection occurs within 30 days after the operation if no implant is left in place or within one year if implant is in place and the infection appears to be related to the operation and infection involves deep soft tissue (e.g. fascia, muscle) of the incision and at least one of the following:

1. Purulent drainage from the deep incision but not from the organ/space component of the surgical site.
2. A deep incision spontaneously dehisces or is deliberately opened by a surgeon when the patient has at least one of the following signs or symptoms: fever (>38°C), localised pain or tenderness, unless incision is culture negative.
3. An abscess or other evidence of infection involving the deep incision is found on direct examination, during reoperation, or by histopathologic or radiologic examination.
4. Diagnosis of deep incisional SSI made by a surgeon or attending physician.

**Organ/Space Surgical Site Infection**

Infection occurs within 30 days after the operation if no implant is left in place or within one year if implant is in place and the infection appears to be related to the operation and infection involves any part of the anatomy (e.g., organs and spaces) other than the incision which was opened or manipulated during an operation and at least one of the following:

1. Purulent drainage from a drain that is placed through a stab wound into the organ/space.
2. Organisms isolated from an aseptically obtained culture of fluid or tissue in the organ/space
3. An abscess or other evidence of infection involving the organ/space that is found on direct examination, during reoperation, or by histopathologic or radiologic examination.
4. Diagnosis of organ/space SSI made by a surgeon or attending physician.

For peer review only

**Table 2: Recruitment centres and estimated recruitment based on number of surgeries performed annually. The hospitals are already recruiting patients.**

| Centre | Estimated n | Estimated % |
|---|---|---|
| Danderyd Hospital | 8000 | 18% |
| Hässleholm/Kristianstad | 10000 | 22% |
| Huddinge Hospital | 6000 | 13% |
| Akademiska Hospital | 10000 | 22% |
| Löwenströmska | 3000 | 7% |
| Umeå | 8000 | 18% |
| **Total sample size** | **45000** | **100%** |

For peer review only

# Appendix 1

## *1 R-script power analysis*

```
# A certain number of infections in the dataset will not be due
# to the surgery that will be adding noise ot the calcualtion.
# We estimate this to 2% based on previous drug registry study experience
unrelated_rate <- 2/100
sig_lvl <- 0.05 # 0.05 with multiplicity for interim analysis of 0.005 added

##############################################
# Power calculation for all regular theaters #
##############################################
# The effect size in a regular operating theater
efx_size <- 0.4
# Base rate of SSI
inf.rate_base <- 2/100
# The SSI rate after the effect
inf.rate_efx <- inf.rate_base*(1-efx_size)
# The power is a combination of the unrelated rate + the rates of interest
power.prop.test(p1 = unrelated_rate + inf.rate_base,
                p2 = unrelated_rate + inf.rate_efx,
                sig.level = sig_lvl,
                power = 0.8)


##########################################
# Power calculation for a combination of #
# regular and ultra-clean theaters       #
##########################################
# The reduced effect in ultra-clean environments
efx_size_clean <- 0.25
# The expected infection rate in the ultraclean operating theaters
inf.rate_clean <- inf.rate_base * 0.8
# The proportion of ultra-clean operating theaters
prop_clean <- 0.8
# The SSI rate when combining the effect in regular and ultraclean operating theaters
inf.mix <- inf.rate_base * (1-prop_clean) +
  inf.rate_clean * prop_clean
# The reduced rate for above combination
inf.mix_efx <- inf.rate_base *
  (1-prop_clean) *
  (1-efx_size) +
  inf.rate_clean *
  prop_clean *
  (1-efx_size_clean)
# The power is a combination of the unrelated rate + the rates of interest
power.prop.test(p1 = unrelated_rate + inf.mix,
                p2 = unrelated_rate + inf.mix_efx,
                sig.level = sig_lvl,
                power = 0.8)
```

# Appendix 2

***Informed consent material (freely translated from Swedish)***

Since December 2016, there is an ongoing randomized trial at the XXXX Hospital, in which all patients that undergo orthopaedic surgery are included. The trial is performed to evaluate a new technique for air-purification in the operating rooms. We will perform follow-ups of all patients treated with surgery in those operating rooms, to check if the new air-purification has reduced the infection rate. Due to the large number of included patients in the study (approx. 45 000), the only data compiled for analysis will be the one already collected for mandatory national registries. If you had surgery performed at our department during this period of time, and wish more information regarding this study, or if you wish not to be included in the analysis part of the study, please contact any of the persons listed below:

| | | |
|---|---|---|
| Anders Persson | Max Gordon | Olof Sköldenberg |
| e-mail: | e-mail: | e-mail: |
| tel: | tel: | tel: |
| role: | role: | role: |

For peer review only

STANDARD PROTOCOL ITEMS: RECOMMENDATIONS FOR INTERVENTIONAL TRIALS

SPIRIT 2013 Checklist: Recommended items to address in a clinical trial protocol and related documents*

| Section/item | Item No | Description | Addressed on page number |
|---|---|---|---|
| **Administrative information** | | | |
| Title | 1 | Descriptive title identifying the study design, population, interventions, and, if applicable, trial acronym | 1 |
| Trial registration | 2a | Trial identifier and registry name. If not yet registered, name of intended registry | 2 |
| | 2b | All items from the World Health Organization Trial Registration Data Set | |
| Protocol version | 3 | Date and version identifier | |
| Funding | 4 | Sources and types of financial, material, and other support | 14 |
| Roles and responsibilities | 5a | Names, affiliations, and roles of protocol contributors | 1, 14 |
| | 5b | Name and contact information for the trial sponsor | 1 |
| | 5c | Role of study sponsor and funders, if any, in study design; collection, management, analysis, and interpretation of data; writing of the report; and the decision to submit the report for publication, including whether they will have ultimate authority over any of these activities | 10, 14 |
| | 5d | Composition, roles, and responsibilities of the coordinating centre, steering committee, endpoint adjudication committee, data management team, and other individuals or groups overseeing the trial, if applicable (see Item 21a for data monitoring committee) | |

For peer review only

| | | | |
|---|---|---|---|
| **Introduction** | | | |
| Background and rationale | 6a | Description of research question and justification for undertaking the trial, including summary of relevant studies (published and unpublished) examining benefits and harms for each intervention | _____4_______ |
| | 6b | Explanation for choice of comparators | _____4_______ |
| Objectives | 7 | Specific objectives or hypotheses | _____5_______ |
| Trial design | 8 | Description of trial design including type of trial (eg, parallel group, crossover, factorial, single group), allocation ratio, and framework (eg, superiority, equivalence, noninferiority, exploratory) | _____5_______ |
| **Methods: Participants, interventions, and outcomes** | | | |
| Study setting | 9 | Description of study settings (eg, community clinic, academic hospital) and list of countries where data will be collected. Reference to where list of study sites can be obtained | _____6_______ |
| Eligibility criteria | 10 | Inclusion and exclusion criteria for participants. If applicable, eligibility criteria for study centres and individuals who will perform the interventions (eg, surgeons, psychotherapists) | _____6_______ |
| Interventions | 11a | Interventions for each group with sufficient detail to allow replication, including how and when they will be administered | _____6_______ |
| | 11b | Criteria for discontinuing or modifying allocated interventions for a given trial participant (eg, drug dose change in response to harms, participant request, or improving/worsening disease) | _____________ |
| | 11c | Strategies to improve adherence to intervention protocols, and any procedures for monitoring adherence (eg, drug tablet return, laboratory tests) | _____6_______ |
| | 11d | Relevant concomitant care and interventions that are permitted or prohibited during the trial | _____________ |
| Outcomes | 12 | Primary, secondary, and other outcomes, including the specific measurement variable (eg, systolic blood pressure), analysis metric (eg, change from baseline, final value, time to event), method of aggregation (eg, median, proportion), and time point for each outcome. Explanation of the clinical relevance of chosen efficacy and harm outcomes is strongly recommended | _____6,7_____ |
| Participant timeline | 13 | Time schedule of enrolment, interventions (including any run-ins and washouts), assessments, and visits for participants. A schematic diagram is highly recommended (see Figure) | _____6,7_____ |



For peer review only - http://bmjopen.bmj.com/site/about/guidelines.xhtml

| | | | |
|---|---|---|---|
| Sample size | 14 | Estimated number of participants needed to achieve study objectives and how it was determined, including clinical and statistical assumptions supporting any sample size calculations | _____8_______ |
| Recruitment | 15 | Strategies for achieving adequate participant enrolment to reach target sample size | _____9_______ |

**Methods: Assignment of interventions (for controlled trials)**

Allocation:

| | | | |
|---|---|---|---|
| Sequence generation | 16a | Method of generating the allocation sequence (eg, computer-generated random numbers), and list of any factors for stratification. To reduce predictability of a random sequence, details of any planned restriction (eg, blocking) should be provided in a separate document that is unavailable to those who enrol participants or assign interventions | _____10______ |
| Allocation concealment mechanism | 16b | Mechanism of implementing the allocation sequence (eg, central telephone; sequentially numbered, opaque, sealed envelopes), describing any steps to conceal the sequence until interventions are assigned | _____10______ |
| Implementation | 16c | Who will generate the allocation sequence, who will enrol participants, and who will assign participants to interventions | _____10______ |
| Blinding (masking) | 17a | Who will be blinded after assignment to interventions (eg, trial participants, care providers, outcome assessors, data analysts), and how | _____10______ |
| | 17b | If blinded, circumstances under which unblinding is permissible, and procedure for revealing a participant's allocated intervention during the trial | _____________ |

**Methods: Data collection, management, and analysis**

| | | | |
|---|---|---|---|
| Data collection methods | 18a | Plans for assessment and collection of outcome, baseline, and other trial data, including any related processes to promote data quality (eg, duplicate measurements, training of assessors) and a description of study instruments (eg, questionnaires, laboratory tests) along with their reliability and validity, if known. Reference to where data collection forms can be found, if not in the protocol | _____11______ |
| | 18b | Plans to promote participant retention and complete follow-up, including list of any outcome data to be collected for participants who discontinue or deviate from intervention protocols | _____________ |

| | | | |
|---|---|---|---|
| Data management | 19 | Plans for data entry, coding, security, and storage, including any related processes to promote data quality (eg, double data entry; range checks for data values). Reference to where details of data management procedures can be found, if not in the protocol | ______12______ |
| Statistical methods | 20a | Statistical methods for analysing primary and secondary outcomes. Reference to where other details of the statistical analysis plan can be found, if not in the protocol | ______12______ |
| | 20b | Methods for any additional analyses (eg, subgroup and adjusted analyses) | ______12______ |
| | 20c | Definition of analysis population relating to protocol non-adherence (eg, as randomised analysis), and any statistical methods to handle missing data (eg, multiple imputation) | ______12______ |
| **Methods: Monitoring** | | | |
| Data monitoring | 21a | Composition of data monitoring committee (DMC); summary of its role and reporting structure; statement of whether it is independent from the sponsor and competing interests; and reference to where further details about its charter can be found, if not in the protocol. Alternatively, an explanation of why a DMC is not needed | ______13______ |
| | 21b | Description of any interim analyses and stopping guidelines, including who will have access to these interim results and make the final decision to terminate the trial | ______14______ |
| Harms | 22 | Plans for collecting, assessing, reporting, and managing solicited and spontaneously reported adverse events and other unintended effects of trial interventions or trial conduct | ______________ |
| Auditing | 23 | Frequency and procedures for auditing trial conduct, if any, and whether the process will be independent from investigators and the sponsor | ______________ |
| **Ethics and dissemination** | | | |
| Research ethics approval | 24 | Plans for seeking research ethics committee/institutional review board (REC/IRB) approval | ______14______ |
| Protocol amendments | 25 | Plans for communicating important protocol modifications (eg, changes to eligibility criteria, outcomes, analyses) to relevant parties (eg, investigators, REC/IRBs, trial participants, trial registries, journals, regulators) | ______________ |

| | | | |
|---|---|---|---|
| Consent or assent | 26a | Who will obtain informed consent or assent from potential trial participants or authorised surrogates, and how (see Item 32) | _____14______ |
| | 26b | Additional consent provisions for collection and use of participant data and biological specimens in ancillary studies, if applicable | ______________ |
| Confidentiality | 27 | How personal information about potential and enrolled participants will be collected, shared, and maintained in order to protect confidentiality before, during, and after the trial | ______________ |
| Declaration of interests | 28 | Financial and other competing interests for principal investigators for the overall trial and each study site | _____14______ |
| Access to data | 29 | Statement of who will have access to the final trial dataset, and disclosure of contractual agreements that limit such access for investigators | _____12______ |
| Ancillary and post-trial care | 30 | Provisions, if any, for ancillary and post-trial care, and for compensation to those who suffer harm from trial participation | ______________ |
| Dissemination policy | 31a | Plans for investigators and sponsor to communicate trial results to participants, healthcare professionals, the public, and other relevant groups (eg, via publication, reporting in results databases, or other data sharing arrangements), including any publication restrictions | _____14______ |
| | 31b | Authorship eligibility guidelines and any intended use of professional writers | ______________ |
| | 31c | Plans, if any, for granting public access to the full protocol, participant-level dataset, and statistical code | ______________ |
| **Appendices** | | | |
| Informed consent materials | 32 | Model consent form and other related documentation given to participants and authorised surrogates | _____18______ |
| Biological specimens | 33 | Plans for collection, laboratory evaluation, and storage of biological specimens for genetic or molecular analysis in the current trial and for future use in ancillary studies, if applicable | ______________ |

*It is strongly recommended that this checklist be read in conjunction with the SPIRIT 2013 Explanation & Elaboration for important clarification on the items. Amendments to the protocol should be tracked and dated. The SPIRIT checklist is copyrighted by the SPIRIT Group under the Creative Commons "Attribution-NonCommercial-NoDerivs 3.0 Unported" license.



For peer review only - http://bmjopen.bmj.com/site/about/guidelines.xhtml

BMJ Open

# Study protocol: The EPOS-trial - The effect of air-filtration through a plasma chamber on the incidence of surgical site infection in orthopaedic surgery. A randomized, double-blind, placebo-controlled trial

| | |
|---|---|
| Journal: | *BMJ Open* |
| Manuscript ID | bmjopen-2020-047500.R1 |
| Article Type: | Protocol |
| Date Submitted by the Author: | 06-Jun-2021 |
| Complete List of Authors: | Persson, Anders; Karolinska Institutet Institutionen för kliniska vetenskaper Danderyds sjukhus,<br>Atroshi, Isam; Lund University, Department of Clinical Sciences; Hässleholm-Kristianstad Hospitals, Department of Orthopaedics<br>Tyszkiewicz, Thomas; Lund University, Department of Clinical Sciences; Hässleholm-Kristianstad Hospitals, Department of Orthopaedics<br>Hailer, Nils; Uppsala University, Department of Surgical Sciences; Uppsala University Hospital, Department of Orthopaedics<br>Lazarinis, Stergios; Uppsala University, Department of Surgical Sciences; Uppsala University Hospital, Department of Orthopaedics<br>Eisler, Thomas; Karolinska Institutet Institutionen för kliniska vetenskaper Danderyds sjukhus<br>Brismar, Harald; Karolinska Institute Department of Clinical Science Intervention and Technology; Karolinska Universitetsjukhuset i Huddinge, Department of Orthopaedics and Biotechnology<br>Mukka, Sebastian; Umea University, Department of Surgical and Perioperative Sciences; Umeå University Hospital<br>Kernell, Per-Juan; Löwenströmska Hospital, GHP Ortho Center Stockholm<br>Mohaddes, Maziar; Swedish Hip Arthroplasty Register; Institute of Clinical Sciences. Sahlgrenska Academy. University Of Gothenburg, Department of Orthopaedics<br>Sköldenberg, Olof; Karolinska Institutet Institutionen för kliniska vetenskaper Danderyds sjukhus<br>Gordon, Max; Karolinska Institutet Institutionen för kliniska vetenskaper Danderyds sjukhus |
| <b>Primary Subject Heading</b>: | Infectious diseases |
| Secondary Subject Heading: | Surgery |
| Keywords: | Orthopaedic & trauma surgery < SURGERY, Infection control < INFECTIOUS DISEASES, Hip < ORTHOPAEDIC & TRAUMA SURGERY, Adult orthopaedics < ORTHOPAEDIC & TRAUMA SURGERY, Knee < ORTHOPAEDIC & TRAUMA SURGERY |
| | |

SCHOLARONE™
Manuscripts

# Study protocol: The EPOS-trial - The effect of air-filtration through a plasma chamber on the incidence of surgical site infection in orthopaedic surgery. A randomized, double-blind, placebo-controlled trial

Anders Persson[1], Isam Atroshi[2], Thomas Tyszkiewicz[2], Nils Hailer[3], Stergios Lazarinis[3], Thomas Eisler[1], Harald Brismar[4], Sebastian Mukka[5], Per-Juan Kernell[6], Maziar Mohaddes[7], Olof Sköldenberg[1], Max Gordon[1]

1. Karolinska Institutet, Department of Clinical Sciences, Danderyd Hospital, Division of Orthopaedics, Stockholm, Sweden
2. Lund University, Department of Clinical Sciences, Orthopaedics, Lund, and Department of Orthopaedics, Hässleholm-Kristianstad Hospitals, Kristianstad, Sweden
3. Uppsala University, Department of Surgical  Sciences, Orthopaedics, Uppsala University Hospital, Uppsala, Sweden
4. Karolinska Institutet, Department of Clinical Science, Intervention and Technology, Department of Orthopaedics and Biotechnology, Karolinska University Hospital in Huddinge, Stockholm, Sweden
5. Umeå University, Department of Surgical and Perioperative Sciences, Umeå University Hospital, Umeå, Sweden
6. GHP Ortho Center Stockholm, Löwenströmska Hospital
7. Department of Orthopaedics, Institute of Clinical Sciences, Sahlgrenska Academy, Gothenburg University, Gothenburg, Sweden, and The Swedish Arthroplasty Register, Gothenburg, Sweden

Correspondance: Anders Persson, M.D., PhD student, Department of Clinical Sciences, Dep. of Orthopaedics, Karolinska Institutet at Danderyd Hospital, S-18288 Danderyd, Sweden, tel: +46 8 12355000

E-mail: anders.persson@sll.se

**Key words:** surgical site infection, prevention, RCT, air-filtration, operating room, environment

**Word count:** 2998

For peer review only

# Abstract

### Introduction

There is controversy regarding the importance of air-transmitted infections for surgical site infections (SSI's) after orthopaedic surgery. Research has been hindered by both the inability in blinding the exposure, and by the need for recruiting large enough cohorts. The aim of this study is to investigate whether using a new form of air-purifier using plasma-air-purification (PAP) in operating rooms (OR's) lower the SSI-rate, or not.

### Methods and analysis

Multicenter, double-blind, cluster-randomized, placebo-controlled trial conducted at seven hospitals 2017-2022. All patients that undergo orthopaedic surgery for minimum 30 minutes are included. Intervention group: patients operated in OR with PAP-devices turned ON. Control group: patients operated in OR with PAP-devices turned OFF. Randomization: Each OR will be randomized in periods of 4, 6, or 8 weeks to either have the devices on or off. Primary outcome: Any SSI postoperatively defined as a composite endpoint of any of the following: use of isoxazolylpenicillin, clindamycin or rifampicin for 2 days or more, ICD codes or Nordic Medico-Statistical Committee (NOMESCO) codes indicating postoperative infection. In a second step, we will perform a chart review on those patients with positive indicators of SSI to further validate the outcome. Secondary outcomes are described in the methods' section. Power: We assume an SSI rate of 2%, an SSI reduction rate of 25% and we need approx. 45 000 patients to attain a power of 80% at a significance level of 0.05.

### Ethics and dissemination

The study is approved by the Swedish Ethical Review Authority. The interim analysis results from the study will be presented only to the researchers involved unless the study thereafter is interrupted for whatever reason. Publication in a medical journal will be presented after inclusion of the last patient.

### Registration details

The EPOS trial is registered at ClinicalTrials.gov with identifier NCT02695368.


For peer review only

## Strengths and limitations of this study

- ✓ EPOS is a multicenter, placebo-controlled trial with approximately 45 000 study subjects, that will evaluate the effect of plasma-air-purification on the incidence of surgical site infections after orthpaedic surgery.
- ✓ The double-blinded design provides strong internal validity to the study results.
- ✓ The cluster-randomization design, which is created through switching of the exposure within each operating room, minimizes the risk of allocation bias.
- ✓ This study is the first RCT to our knowlegde, investigating the true cause-and effect relationship between an air-purifying intervention in operating rooms and surgical site infections.
- ✓ The primary limitation to the study is the resource intensity, mainly due to the large number of study subjects required to study such an unusual outcome, and the concomitant review of medical records to validate the outcome.

For peer review only



For peer review only - http://bmjopen.bmj.com/site/about/guidelines.xhtml

## Introduction

Despite surgery in clean OR's, surface sterilization, and antibiotics, SSI after orthopaedic surgery have an overall estimated incidence of 1-4% (1-3). This feared complication is associated with long-term antibiotics, repeated surgeries, prolonged hospital stays, economic burden, and a poorer end result for individual patients (4, 5). Prevention of SSI's is therefore of paramount importance.

Air flow within the OR can spread airborne particles, posing a potential risk for postoperative infection. These airborne particles include dust, textile fibres, skin scales, and respiratory aerosols, loaded with viable microorganisms (including *Staphylococcus aureus*) having been released mainly from the surgical team members and patient into the surrounding air of the OR. These particles have been shown to settle onto surfaces including the surgical wound and instruments (6). Thus, air-transmitted infections is one of the main reasons for SSI (6), making this an interesting intervention target.

Ever since the ground-breaking study by Charnley in 1969 (7), which showed that cleaner air in the OR drastically improved infection rates after total hip arthroplasty, vast efforts have been made to address this issue. Lidwell carried on his work, showing that ultra-clean air (i.e. <10 colony-forming units (CFU)/$m^3$) in combination with body-exhaust suits and prophylactic antibiotics further improved infection rates (8-10). Based on their work, in combination with several reports showing that laminar air flow (LAF) reduces bacterial contamination in the OR air (11-13), many OR's are today equipped with LAF ventilation. Unfortunately though, when evaluated in large cohort studies, systematic reviews and meta-analyses, LAF systems in modern state-of-the-art OR's have so far failed to prove efficient in preventing infections, compared to conventional ventilation (14-19).

The plasma air-purification (PAP) system used in our current study is an air purifier that sterilizes the air particles through a plasma chamber (Figure 1). Air in the OR is pumped through the chamber, and by using a small current it transforms the air in the vicinity of the electrode into plasma, which eradicates any bacteria that pass through (Figure 2). The small size of the machine allows it to fit into any operating theatre without interfering with existing equipment (20).

Similar systems exists that use ultraviolet light to sterilize air particles and reduce the rate of hospital acquired infections (21, 22). There are though few peer-reviewed articles regarding air-purification. While we have not found any randomized clinical trials, there are randomised field trials, outside hospital settings, that have shown positive effects in vivo. In a blinded randomized field trial on healthy volunteers using air purifiers, a significant reduction in air-particles were seen and this also led to a reduction of stress hormone for the participants. (23)

In hospital and OR settings the PAP technology alone significantly reduces the number of colony-forming units (CFUs) of staphylococci (the most common infecting microorganism in SSI) from 49-97% (24). In non-randomized studies it has been shown to reduce respiratory infections, personnel sick-leave, and severe infectious outbreaks (22, 24). These effects have though not been validated in randomized clinical trials in operating room settings. However, this is also true for all methods of reducing air-borne pathogens in operating rooms

For peer review only

that currently are used, such as surgery in LAF OR’s. The need for Level 1 evidence in this field of medicine is urgent.

The EPOS-trial is therefore designed as a multicentre, double-blinded, cluster-randomized, placebo-controlled trial.

The aim of this study is to investigate whether using PAP devices that clean the air from contaminating particles in OR’s lower the rate of SSI, or not. The primary endpoint will be SSI rate within 12 weeks postoperatively, defined as either use of antibiotics targeting common implant pathogens, ICD code or NOMESCO code indicating postoperative infection. This proxy variable is more closely described in the methods section. We hypothesize that PAP can reduce the incidence of SSI in orthopaedic surgery by 25%. Secondary aims include investigating the number of prescribed antibiotics as well as the number of needed readmissions and length-of-stay for SSI.

For peer review only

# Methods: Participants, interventions and outcomes

### *Study setting*

The study is being conducted at seven major hospitals in Sweden (Table 2). Inclusion will take place between April 2017 and 31st of Dec 2021. The randomized clinical trial setting has been chosen to control for the huge number of possible confounders influencing the outcome. The study protocol has been written according to the SPIRIT (Standard Protocol Items for Randomized Trials) statement. A CONSORT flow diagram, published as a supplementary file to this protocol, describes our study graphically.

### *Eligibility criteria*

We will **include** (1) all patients that undergo surgery for 30 minutes or longer at each centre during the study period. We assume that surgeries lasting less than 30 minutes are less susceptible to SSI's. Including those in this study would therefore result in a larger cohort. We will **exclude** surgeries on 1) already infected surgical sites, defined as: ICD- or NOMESCO codes indicating infection (same as those used for the primary outcome, see below), open fractures, traumatic wounds, vacuum assisted wound therapy, 2) patients that have withdrawn antibiotics 2 weeks or less prior to surgery, and 3) patients that have actively marked their hospital charts with an added privacy notice. If patients have multiple surgeries during the study period, only the first operation will be included.

### *Intervention*

At each hospital, all OR's that perform surgery on orthopaedic patients will be equipped with three (3) PAP-systems each. The groups are defined as: **Intervention group:** those operated where the PAP device has been turned on for at least 2 days prior to index surgery. **Control group:** those where the PAP device has been turned off for at least 2 days prior to index surgery. **Mixed group:** those receiving surgeries in OR's within 2 days after the PAP device switches status. We will also in the analysis sub-group the study subjects according to measurements prior to study start into: ***Regular*** operating rooms (≥10 CFU/ $m^3$) and ***Ultra-clean*** operating rooms (<10 CFU/$m^3$).

PAP device status and function will be monitored continuously during the inclusion period through standardized manual controls every 3 months, and also at the end of the inclusion period by validating PAP device status retrospectively through memory card recordings in each device.

### *Outcomes*

The **primary outcome** is any indication of SSI within 12 weeks postoperatively, defined as a composite endpoint of any of the following:

1. Withdrawal or other documented use of antibiotics corresponding to 2 days or more after surgery targeting Staphylococcus aureus
2. ICD code indicating postoperative infection (at date of readmission)
3. NOMESCO code indicating postoperative infection


For peer review only - http://bmjopen.bmj.com/site/about/guidelines.xhtml

In a second step, we will perform a chart review on those patients with positive indicators of SSI to further validate the outcome. We will use the internationally accepted CDC definition of SSI when finally establishing that a SSI has occurred (Table 1) (25).

The **effect size** of the primary endpoint is the relative risk of contracting SSI for the intervention group versus the control group and will be calculated by dividing the probability of contracting a SSI in the intervention group by the control group = $\frac{SSI\ rate\ intervention\ group}{SSI\ rate\ control\ group}$.
The **effect size** will also be presented as an absolute risk difference = $SSI\ rate\ intervention\ group - SSI\ rate\ control\ group.$

The primary outcome is a **surrogate variable** for SSI as it due to the large sample size is practically impossible to have all patients come back for outpatient visits and be visually inspected. To further validate the outcome, a medical record review will be performed in a second step on all individual patients with indication of SSI. Our expectations are that the choice and succeeding validation of this proxy variable can provide us with a reliable tool for investigating SSI's in future projects.

The Swedish health-care registers, especially the Prescribed Drug Register and the Patient Register will make sure that we get an almost complete (>99%) coverage of all relevant SSI's.

The **secondary outcomes** are:

a) Withdrawal or other documented use of any antibiotics for 2 days or more after surgery during the first 30 postoperative days
b) Number of days with antibiotics during the first 30 days
c) Same as a) & b) but up to 90 days after surgery
d) Death during the first 2 postoperative years

These analyses will also be performed with and without adjustment for pre-operative antibiotic use 6 months prior to the surgery.

### *Sample size*

The re-operation rate in Sweden due to infections within the first 2 years after surgery is 1.3% (3) for primary total hip replacements (THR's). This does not include THR's in fracture patients and other types of surgeries that are more susceptible to infections. We therefore assume that the SSI rate in our study population is 2% (1-3). Similarly, we know from other data that about 0.7% withdraw the antibiotics associated with the primary outcome within a 3 months' period prior to surgery. To account for infections unrelated to surgery we assume that the infection noise rate, i.e. non-surgical site infections, is less than 2%.
***Multicentre power with ultra-clean air:*** Hospitals with OR's with ultra-clean air may be less susceptible to the effect with an already lower infection rate. Pre-study CFU measurements suggest that approximately 80% of the included OR's are ultra-clean. If we assume that the infection rate is 2% or less, and that the effect size is reduced to 25% in an ultra-clean environment, we will need to recruit 22,630 patients in each group, i.e. approximately 45,000 patients to attain a power of 80% with a significance level of 0.05. This will be our target population. We expect a very low drop-out rate/missing data in the study, estimated to be <1%. At the interim analysis the p-value will be set 10 times lower; at 0.005; i.e. if a statistically significant result between the groups is observed at 18 months, the study will be


For peer review only - http://bmjopen.bmj.com/site/about/guidelines.xhtml

stopped. See Figure 3 and Appendix 1 for details in R and explanation of the script for the sample size analysis.

### *Recruitment*

We estimate that with only one center, such as Danderyd Hospital, recruitment would take >5 years. The hospital operates about 4,500 patients each year and as it is unlikely that more than 500 of these will be excluded. We therefore anticipate recruiting 4,000 patients/year. Performing this study in a multi-center setting is therefore crucial. In Table 2 the participating centers are presented, and their estimated proportion of patients included.

### *Patient and Public Involvement*

Representatives of the Swedish Osteoporosis Society (www.osteoporos.org) and the Swedish Rheumatic Society (www.reumatiker.se) are members of the study steering committee, and participate mainly in discussions regarding plans for reporting and publishing the results of the study.

For peer review only



For peer review only - http://bmjopen.bmj.com/site/about/guidelines.xhtml

## Methods: Assignment of interventions

### *Allocation*

The three PAP devices in each OR will synchronically be randomized in periods of 4, 6, or 8 weeks to either have the system “on” or “off”. The switch will always occur midnight Friday in order to limit the patients exposed to partial effect during the first two days after switching status. The system can be programmed to be active (i.e. plasma chamber eradicating bacteria) at any given timeframe. The manufacturer of the PAP devices will prepare the randomization allocation and automatic execution of it. The randomization sequence is at minimum 8 years long and will be submitted to a third, independent party, responsible for keeping the allocation secret until interim analyses or study end. At the interim analyses only allocations up to that date will be released.

### *Blinding*

The on/off only refers to the plasma chamber responsible for the antimicrobial effect. As the machine retains the air flow it will be impossible for staff, surgeons and patients to determine the status of the machine from the outside. The device will also automatically switch status where the true status is concealed for all study participants including other hospital personnel until the end of the study.

For peer review only



For peer review only - http://bmjopen.bmj.com/site/about/guidelines.xhtml

# Methods: Data collection, management, and analysis

### *Data collection methods*

For the primary endpoint the following codes will be used to detect if individual patients have contracted an SSI following surgery. If any of these codes indicate SSI, a chart review will be performed in a second step to verify the outcome:

1. From The Swedish Prescribed Drug Register: withdrawal of antibiotics targeting Staphylococcus aureus corresponding to a minimum amount of 2 defined daily dosages (an estimate provided by the registry for the expected daily dosage). The date of withdrawal will serve as an indicator of treatment start unless inpatient data is available with more granular information. The drug ATC codes considered to target relevant bacteria are:
   a. J01CF05 (isoxazolylpenicillin)
   b. J01FF01 (clindamycin)
   c. J04AB02 (rifampicin)
2. From The National Patient Registry: ICD trigger codes indicating postoperative infections:
   a. T793 – Post-traumatic wound infection, not elsewhere classified
   b. T814 – Infection following a procedure, not elsewhere classified
   c. T84[5-7] – Infection and inflammatory reaction due to internal joint prosthesis, internal fixation device, or other internal orthopaedic prosthetic devices, implants and grafts
   d. T874 – Infection of amputation stump
   e. B9[5,6,8] – Bacterial specification
   f. L0[2-4] – Cutaneous abscess, furuncle and carbuncle; cellulitis
   g. A[24]6 – Erysipeloid, erysipelas
3. From The National Patient Registry: NOMESCO trigger codes indicating postoperative infections:
   a. Incision abscess: TN[A-H]05
   b. Surgeries due infections: N[A-H]S[0-4,9]9
   c. Extremities wound revision: Q[CD]B05
   d. Re-operation for infection: N[A-H]W69
   e. Vacuum treatment: DQ023

Both local and national registry data will be used according to availability. For the admission episodes with the code indicators the admission date is the index date, i.e. if an admission occurs after 91 days with a trigger code it will not be considered an indicator of a postoperative infection.

In-hospital information systems will supply information on:

a) Patient ID
b) Date(s) of surgery
c) Surgery associated codes including operated side
d) Operating room
e) In-hospital antibiotics

Both the surgical and medical data records will be retrieved depending on availability. Only centres that can provide the above data will be allowed to participate.


For peer review only - http://bmjopen.bmj.com/site/about/guidelines.xhtml

The Swedish National Patient Register includes all in-patient care and outpatient visits in Sweden with discharge codes according to ICD-10, NOMESCO codes and admission/discharge dates (26).

The Swedish Prescribed Drug Register (PDR) includes any withdrawn prescriptions. Prescriptions that are never withdrawn by patients and drugs bought over the counter without prescriptions are not included. The data fields used were the drug ATC-code, number of pills, and prescription text (27).

### *Data management*

The study data will be securely managed and stored encrypted at a computer within Karolinska Institutet at Danderyd Hospital. No other than the authors stated above will gain access to raw data.

### *Statistical methods*

The primary outcome is a binary variable where there are three groups. We will use logistic regression where the reference group is the placebo group, and the significance is related to the intervention group. The estimate for the mixed group is only for relating dose-effect, i.e. the group will not be pooled with either the placebo or the intervention group. Due to the randomization we do not intend to have any other covariates as confounders in the model. Similar methodology will be applied to the antibiotic's binary outcome. The number of days with antibiotics will be modelled using a linear regression with the similar interpretation to above regarding predictors. Mortality will be modelled using a cox proportional hazards model with time since surgery calculated as time to death, migration or 2 years. The cox model will not contain any covariates due to the randomized study nature. The analysis will be performed by an epidemiologist/statistician in our team (MG) and will be performed as a per-protocol analysis.

We will handle confounding by including only the patient's 1st surgical procedure. The randomization process will handle other confounding such as confounding by selection. Procedural confounding will be handled by the external part who has done the randomization procedure for each PAP device. Regarding dropouts the health-care registers and chart review done in the study will ensure a very low (<1%) drop-out rate. Individual patients can also request that they are excluded from the registers and thus from the study, but by experience, this is very rare and will also not affect our ability to analyse our primary and secondary endpoints.



For peer review only - http://bmjopen.bmj.com/site/about/guidelines.xhtml

# Methods: Monitoring

### *Data monitoring*

At 12 months after study start (2018-04-17) an interim analysis will be performed and the recruitment rate from each center will be evaluated. The study recruitment will end once we have reached a minimum of 45,000 patients. During the 2nd half of 2017/early 2018, the data quality of each recruiting center will be evaluated by extracting data from each center's hospital information center.

For peer review only

## Ethics and dissemination

The study is being conducted in accordance with the ethical principles of the Helsinki Declaration, and is approved by the Swedish Ethical Review Authority (2015/1139-31/4).

The interim analysis results from the study will be presented only to the researchers involved unless the study thereafter is interrupted due to significant difference in infection rate between the two groups. Publication in a medical journal will be presented after inclusion of the last patient.

Study participant information will be published on the hospital web site (see Appendix 2). Due to feasibility reasons in a study with approximately 45 000 study participants, and the very low probability of any adverse effects related to the intervention, no personal consent forms will be collected. However, individual patients can request exclusion from the data analysis.

The EPOS trial is registered at ClinicalTrials.gov with identifier NCT02695368.

## Author contributions

AP operates the trial, and led the writing of this manuscript, with contributions from the rest of the authors. TE, MM, HB, NH, SL, TT, IA, SM and PK operate the inclusion centers. MG and OS designed the original study and developed the protocol. MG is the responsible statistician and supervises the study. All authors contributed to the editing and redrafting of the manuscript.

## Funding statement

8.8 million SEK from the Swedish Research Council has been received for 2017-2020 (grant number 2017-00198). 1.4 million SEK from ALF (Stockholm County and Karolinska Institute, application number 20160251, record number LS 2015-1198) has been received for 2017-2018. We have a discounted price for the rental of the machines but have chosen not to apply for funding from the manufacturers of the plasma-air-purifier equipment to ensure that the study is independent.

## Competing interests statement

None.

For peer review only

## Figures

1. Rendered view of Novaerus NV800
2. Air-flow through the air-purifier
3. Graphs showing the impact of base SSI rate and rate of unrelated infections on the required sample size

For peer review only

## References

1. Reilly J, Allardice G, Bruce J, Hill R, McCoubrey J. Procedure-specific surgical site infection rates and postdischarge surveillance in Scotland. Infection control and hospital epidemiology. 2006;27(12):1318-23.
2. Pull ter Gunne AF, Cohen DB. Incidence, prevalence, and analysis of risk factors for surgical site infection following adult spinal surgery. Spine. 2009;34(13):1422-8.
3. Lindgren JV, Gordon M, Wretenberg P, Kärrholm J, Garellick G. Validation of reoperations due to infection in the Swedish Hip Arthroplasty Register. BMC musculoskeletal disorders. 2014;15:384.
4. de Lissovoy G, Fraeman K, Hutchins V, Murphy D, Song D, Vaughn BB. Surgical site infection: incidence and impact on hospital utilization and treatment costs. Am J Infect Control. 2009;37(5):387-97.
5. Bozic KJ, Katz P, Cisternas M, Ono L, Ries MD, Showstack J. Hospital resource utilization for primary and revision total hip arthroplasty. J Bone Joint Surg Am. 2005;87(3):570-6.
6. Fernstrom A, Goldblatt M. Aerobiology and its role in the transmission of infectious diseases. Journal of pathogens. 2013;2013:493960.
7. Charnley J, Eftekhar N. Postoperative infection in total prosthetic replacement arthroplasty of the hip-joint. With special reference to the bacterial content of the air of the operating room. Br J Surg. 1969;56(9):641-9.
8. Lidwell OM, Elson RA, Lowbury EJ, Whyte W, Blowers R, Stanley SJ, et al. Ultraclean air and antibiotics for prevention of postoperative infection. A multicenter study of 8,052 joint replacement operations. Acta orthopaedica Scandinavica. 1987;58(1):4-13.
9. Lidwell OM, Lowbury EJ, Whyte W, Blowers R, Stanley SJ, Lowe D. Airborne contamination of wounds in joint replacement operations: the relationship to sepsis rates. J Hosp Infect. 1983;4(2):111-31.
10. Lidwell OM, Lowbury EJ, Whyte W, Blowers R, Stanley SJ, Lowe D. Effect of ultraclean air in operating rooms on deep sepsis in the joint after total hip or knee replacement: a randomised study. British medical journal (Clinical research ed). 1982;285(6334):10-4.
11. Hirsch T, Hubert H, Fischer S, Lahmer A, Lehnhardt M, Steinau HU, et al. Bacterial burden in the operating room: impact of airflow systems. Am J Infect Control. 2012;40(7):e228-32.
12. Diab-Elschahawi M, Berger J, Blacky A, Kimberger O, Oguz R, Kuelpmann R, et al. Impact of different-sized laminar air flow versus no laminar air flow on bacterial counts in the operating room during orthopedic surgery. Am J Infect Control. 2011;39(7):e25-9.
13. Romano F, Milani S, Ricci R, Joppolo CM. Operating Theatre Ventilation Systems and Their Performance in Contamination Control: "At Rest" and "In Operation" Particle and Microbial Measurements Made in an Italian Large and Multi-Year Inspection Campaign. Int J Environ Res Public Health. 2020;17(19).
14. WHO. Global Guidelines for the Prevention of Surgical Site Infection. WHO Guidelines Approved by the Guidelines Review Committee. Geneva2016.
15. Breier AC, Brandt C, Sohr D, Geffers C, Gastmeier P. Laminar airflow ceiling size: no impact on infection rates following hip and knee prosthesis. Infection control and hospital epidemiology. 2011;32(11):1097-102.
16. Gastmeier P, Breier AC, Brandt C. Influence of laminar airflow on prosthetic joint infections: a systematic review. J Hosp Infect. 2012;81(2):73-8.
17. Bischoff P, Kubilay NZ, Allegranzi B, Egger M, Gastmeier P. Effect of laminar airflow ventilation on surgical site infections: a systematic review and meta-analysis. Lancet Infect Dis. 2017;17(5):553-61.
18. McHugh SM, Hill AD, Humphreys H. Laminar airflow and the prevention of surgical site infection. More harm than good? Surgeon. 2015;13(1):52-8.
19. Pasquarella C, Barchitta M, D'Alessandro D, Cristina ML, Mura I, Nobile M, et al. Heating, ventilation and air conditioning (HVAC) system, microbial air contamination and

For peer review only

surgical site infection in hip and knee arthroplasties: the GISIO-SItI Ischia study. Ann Ig. 2018;30(5 Supple 2):22-35.
20. Novaerus Technology 2020 [Available from: https://cmsifyassets-1290.kxcdn.com/nov-production/uploads/asset/1741/attachment/NV_NanoStrike_Brochure_HIRES.pdf.
21. Anis HK, Curtis GL, Klika AK, Piuzzi NS, Otiso J, Richter SS, et al. In-Room Ultraviolet Air Filtration Units Reduce Airborne Particles During Total Joint Arthroplasty. J Orthop Res. 2020;38(2):431-7.
22. Kane D. Study shows reduced HAIs, airborne pathogens with in-room active UV-C system http://www.mcknights.com/marketplace/study-shows-reduced-hais-airborne-pathogens-with-in-room-active-uv-c-system/article/573958/2017 [
23. Li H, Cai J, Chen R, Zhao Z, Ying Z, Wang L, et al. Particulate Matter Exposure and Stress Hormone Levels: A Randomized, Double-Blind, Crossover Trial of Air Purification. Circulation. 2017;136(7):618-27.
24. James E. Independent validation report for Novareus air treatment systems 2014 [Available from: http://sandbox.novaerus.com/wp-content/uploads/2014/03/Independent-Validation-Report-for-Novaerus-NHS.pdf.
25. Horan TC, Gaynes RP, Martone WJ, Jarvis WR, Emori TG. CDC definitions of nosocomial surgical site infections, 1992: a modification of CDC definitions of surgical wound infections. Infection control and hospital epidemiology. 1992;13(10):606-8.
26. The National Patient Register [Internet]. 2019. Available from: https://www.socialstyrelsen.se/en/statistics-and-data/registers/register-information/the-national-patient-register/.
27. Prescribed Drug Register [Internet]. 2019. Available from: https://www.socialstyrelsen.se/statistik-och-data/register/alla-register/lakemedelsregistret/.

For peer review only

# Tables

Table 1. CDC definition of surgical site infection (SSI)

**Superficial Incisional Surgical Site Infection**

Infection within 30 days after the operation and only involves skin and subcutaneous tissue of the incision and at least one of the following:

1. Purulent drainage with or without laboratory confirmation, from the superficial incision.
2. Organisms isolated from an aseptically obtained culture of fluid or tissue from the superficial incision.
3. At least one of the following signs or symptoms of infection: pain or tenderness, localised swelling, redness, or heat and superficial incision is deliberately opened by surgeon, unless incision is culture-negative.
4. Diagnosis of superficial incisional SSI made by a surgeon or attending physician.

**Deep Incisional Surgical Site Infection**

Infection occurs within 30 days after the operation if no implant is left in place or within one year if implant is in place and the infection appears to be related to the operation and infection involves deep soft tissue (e.g. fascia, muscle) of the incision and at least one of the following:

1. Purulent drainage from the deep incision but not from the organ/space component of the surgical site.
2. A deep incision spontaneously dehisces or is deliberately opened by a surgeon when the patient has at least one of the following signs or symptoms: fever (>38°C), localised pain or tenderness, unless incision is culture-negative.
3. An abscess or other evidence of infection involving the deep incision is found on direct examination, during reoperation, or by histopathologic or radiologic examination.
4. Diagnosis of deep incisional SSI made by a surgeon or attending physician.

**Organ/Space Surgical Site Infection**

Infection occurs within 30 days after the operation if no implant is left in place or within one year if implant is in place and the infection appears to be related to the operation and infection involves any part of the anatomy (e.g., organs and spaces) other than the incision which was opened or manipulated during an operation and at least one of the following:

1. Purulent drainage from a drain that is placed through a stab wound into the organ/space.
2. Organisms isolated from an aseptically obtained culture of fluid or tissue in the organ/space
3. An abscess or other evidence of infection involving the organ/space that is found on direct examination, during reoperation, or by histopathologic or radiologic examination.
4. Diagnosis of organ/space SSI made by a surgeon or attending physician.

Table 2. Recruitment centres and estimated recruitment based on number of surgeries performed annually. The hospitals are already recruiting patients.

| Centre | Estimated n | % recruited |
|---|---|---|
| Danderyd Hospital | 8000 | 18% |
| Hässleholm/Kristianstad | 10 000 | 22% |
| Huddinge hospital | 6000 | 13% |
| Akademiska hospital | 10 000 | 22% |
| Ortho Center | 3000 | 7% |
| Umeå | 8000 | 18% |
| **Total sample size** | **45000** | **100%** |



For peer review only - http://bmjopen.bmj.com/site/about/guidelines.xhtml

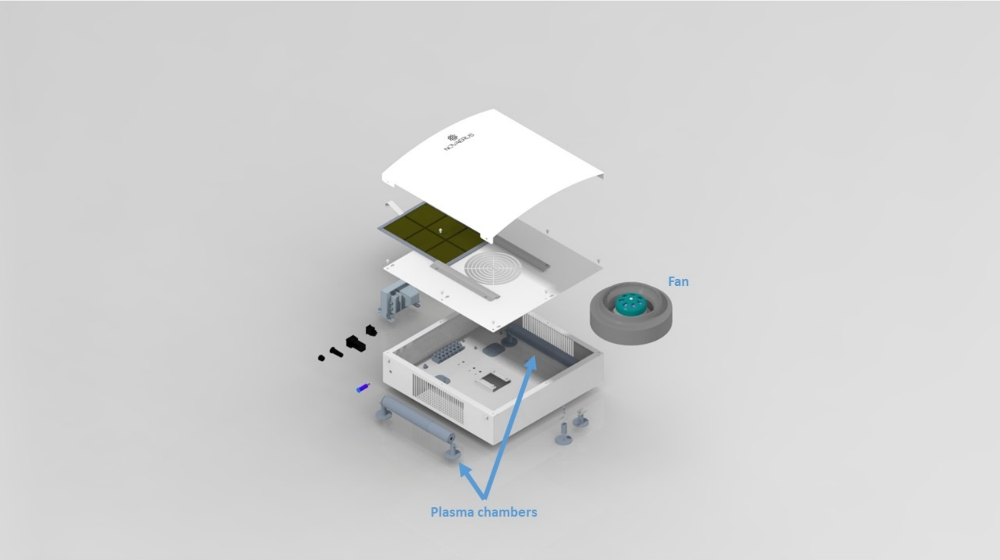


Rendered view of Novaerus NV800

338x190mm (96 x 96 DPI)

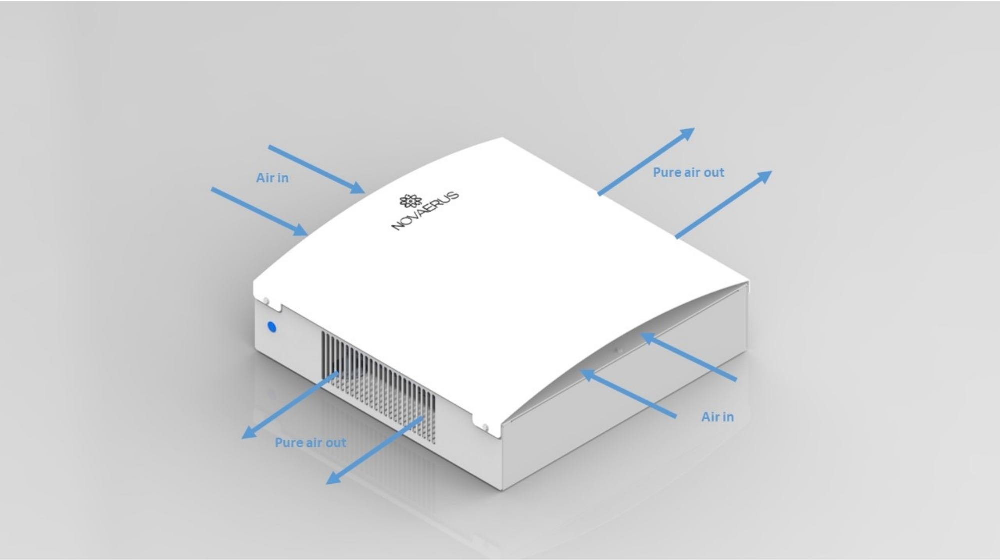


Air flow through the air-purifier

338x190mm (96 x 96 DPI)

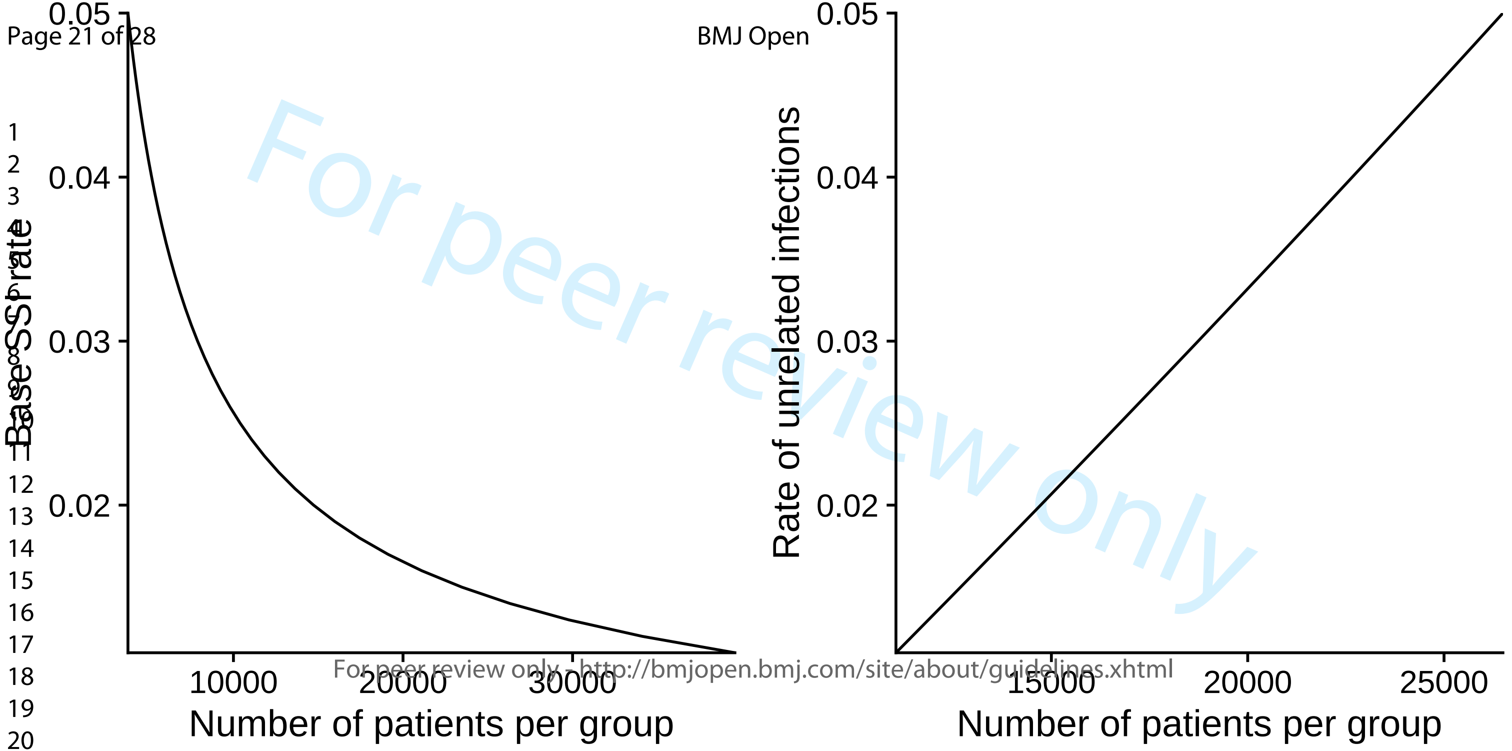


For peer review only

# Appendix 1

## *1 R-script power analysis*

```
# A certain number of infections in the dataset will not be due
# to the surgery that will be adding noise ot the calcualtion.
# We estimate this to 2% based on previous drug registry study experience
unrelated_rate <- 2/100
sig_lvl <- 0.05 # 0.05 with multiplicity for interim analysis of 0.005 added

##############################################
# Power calculation for all regular theaters #
##############################################
# The effect size in a regular operating theater
efx_size <- 0.4
# Base rate of SSI
inf.rate_base <- 2/100
# The SSI rate after the effect
inf.rate_efx <- inf.rate_base*(1-efx_size)
# The power is a combination of the unrelated rate + the rates of interest
power.prop.test(p1 = unrelated_rate + inf.rate_base,
                p2 = unrelated_rate + inf.rate_efx,
                sig.level = sig_lvl,
                power = 0.8)


##########################################
# Power calculation for a combination of #
# regular and ultra-clean theaters       #
##########################################
# The reduced effect in ultra-clean environments
efx_size_clean <- 0.25
# The expected infection rate in the ultraclean operating theaters
inf.rate_clean <- inf.rate_base * 0.8
# The proportion of ultra-clean operating theaters
prop_clean <- 0.8
# The SSI rate when combining the effect in regular and ultraclean operating theaters
inf.mix <- inf.rate_base * (1-prop_clean) +
  inf.rate_clean * prop_clean
# The reduced rate for above combination
inf.mix_efx <- inf.rate_base *
  (1-prop_clean) *
  (1-efx_size) +
  inf.rate_clean *
  prop_clean *
  (1-efx_size_clean)
# The power is a combination of the unrelated rate + the rates of interest
power.prop.test(p1 = unrelated_rate + inf.mix,
                p2 = unrelated_rate + inf.mix_efx,
                sig.level = sig_lvl,
                power = 0.8)
```

# Appendix 2

## *Informed consent material (freely translated from Swedish)*

Since December 2016, there is an ongoing randomized trial at the XXXX Hospital, in which all patients that undergo orthopaedic surgery are included. The trial is performed to evaluate a new technique for air-purification in the operating rooms. We will perform follow-ups of all patients treated with surgery in those operating rooms, to check if the new air-purification has reduced the infection rate. Due to the large number of included patients in the study (approx. 45 000), the only data compiled for analysis will be the one already collected for mandatory national registries. If you had surgery performed at our department during this period of time, and wish more information regarding this study, or if you wish not to be included in the analysis part of the study, please contact any of the persons listed below:

| | | |
|---|---|---|
| Anders Persson | Max Gordon | Olof Sköldenberg |
| e-mail: | e-mail: | e-mail: |
| tel: | tel: | tel: |
| role: | role: | role: |

For peer review only

CONSORT
TRANSPARENT REPORTING of TRIALS

## CONSORT Flow Diagram – Study plan for EPOS

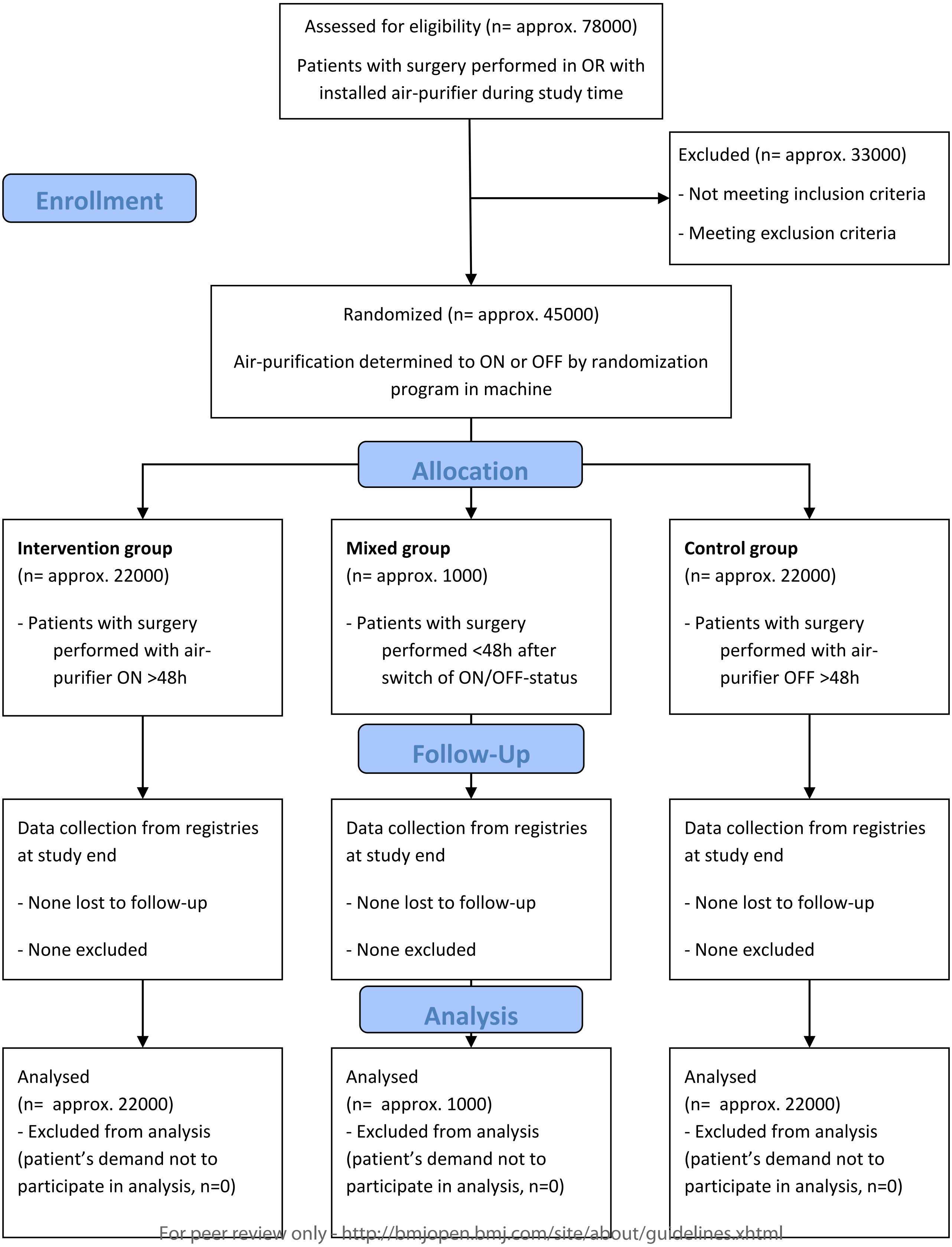


For peer review only - http://bmjopen.bmj.com/site/about/guidelines.xhtml

SPIRIT

STANDARD PROTOCOL ITEMS: RECOMMENDATIONS FOR INTERVENTIONAL TRIALS

SPIRIT 2013 Checklist: Recommended items to address in a clinical trial protocol and related documents*

| Section/item | Item No | Description | Addressed on page number |
|---|---|---|---|
| **Administrative information** | | | |
| Title | 1 | Descriptive title identifying the study design, population, interventions, and, if applicable, trial acronym | 1 |
| Trial registration | 2a | Trial identifier and registry name. If not yet registered, name of intended registry | 2 |
| | 2b | All items from the World Health Organization Trial Registration Data Set | |
| Protocol version | 3 | Date and version identifier | |
| Funding | 4 | Sources and types of financial, material, and other support | 14 |
| Roles and responsibilities | 5a | Names, affiliations, and roles of protocol contributors | 1, 14 |
| | 5b | Name and contact information for the trial sponsor | 1 |
| | 5c | Role of study sponsor and funders, if any, in study design; collection, management, analysis, and interpretation of data; writing of the report; and the decision to submit the report for publication, including whether they will have ultimate authority over any of these activities | 10, 14 |
| | 5d | Composition, roles, and responsibilities of the coordinating centre, steering committee, endpoint adjudication committee, data management team, and other individuals or groups overseeing the trial, if applicable (see Item 21a for data monitoring committee) | |

For peer review only



For peer review only - http://bmjopen.bmj.com/site/about/guidelines.xhtml

**Introduction**

| | | | |
|---|---|---|---|
| Background and rationale | 6a | Description of research question and justification for undertaking the trial, including summary of relevant studies (published and unpublished) examining benefits and harms for each intervention | _____4_______ |
| | 6b | Explanation for choice of comparators | _____4_______ |
| Objectives | 7 | Specific objectives or hypotheses | _____5_______ |
| Trial design | 8 | Description of trial design including type of trial (eg, parallel group, crossover, factorial, single group), allocation ratio, and framework (eg, superiority, equivalence, noninferiority, exploratory) | _____5_______ |

**Methods: Participants, interventions, and outcomes**

| | | | |
|---|---|---|---|
| Study setting | 9 | Description of study settings (eg, community clinic, academic hospital) and list of countries where data will be collected. Reference to where list of study sites can be obtained | _____6_______ |
| Eligibility criteria | 10 | Inclusion and exclusion criteria for participants. If applicable, eligibility criteria for study centres and individuals who will perform the interventions (eg, surgeons, psychotherapists) | _____6_______ |
| Interventions | 11a | Interventions for each group with sufficient detail to allow replication, including how and when they will be administered | _____6_______ |
| | 11b | Criteria for discontinuing or modifying allocated interventions for a given trial participant (eg, drug dose change in response to harms, participant request, or improving/worsening disease) | _____________ |
| | 11c | Strategies to improve adherence to intervention protocols, and any procedures for monitoring adherence (eg, drug tablet return, laboratory tests) | _____6_______ |
| | 11d | Relevant concomitant care and interventions that are permitted or prohibited during the trial | _____________ |
| Outcomes | 12 | Primary, secondary, and other outcomes, including the specific measurement variable (eg, systolic blood pressure), analysis metric (eg, change from baseline, final value, time to event), method of aggregation (eg, median, proportion), and time point for each outcome. Explanation of the clinical relevance of chosen efficacy and harm outcomes is strongly recommended | _____6,7_____ |
| Participant timeline | 13 | Time schedule of enrolment, interventions (including any run-ins and washouts), assessments, and visits for participants. A schematic diagram is highly recommended (see Figure) | _____6,7_____ |


For peer review only - http://bmjopen.bmj.com/site/about/guidelines.xhtml

| | | | |
|---|---|---|---|
| Sample size | 14 | Estimated number of participants needed to achieve study objectives and how it was determined, including clinical and statistical assumptions supporting any sample size calculations | _____8_______ |
| Recruitment | 15 | Strategies for achieving adequate participant enrolment to reach target sample size | _____9_______ |

**Methods: Assignment of interventions (for controlled trials)**

| | | | |
|---|---|---|---|
| Allocation: | | | |
| Sequence generation | 16a | Method of generating the allocation sequence (eg, computer-generated random numbers), and list of any factors for stratification. To reduce predictability of a random sequence, details of any planned restriction (eg, blocking) should be provided in a separate document that is unavailable to those who enrol participants or assign interventions | _____10______ |
| Allocation concealment mechanism | 16b | Mechanism of implementing the allocation sequence (eg, central telephone; sequentially numbered, opaque, sealed envelopes), describing any steps to conceal the sequence until interventions are assigned | _____10______ |
| Implementation | 16c | Who will generate the allocation sequence, who will enrol participants, and who will assign participants to interventions | _____10______ |
| Blinding (masking) | 17a | Who will be blinded after assignment to interventions (eg, trial participants, care providers, outcome assessors, data analysts), and how | _____10______ |
| | 17b | If blinded, circumstances under which unblinding is permissible, and procedure for revealing a participant's allocated intervention during the trial | _____________ |

**Methods: Data collection, management, and analysis**

| | | | |
|---|---|---|---|
| Data collection methods | 18a | Plans for assessment and collection of outcome, baseline, and other trial data, including any related processes to promote data quality (eg, duplicate measurements, training of assessors) and a description of study instruments (eg, questionnaires, laboratory tests) along with their reliability and validity, if known. Reference to where data collection forms can be found, if not in the protocol | _____11______ |
| | 18b | Plans to promote participant retention and complete follow-up, including list of any outcome data to be collected for participants who discontinue or deviate from intervention protocols | _____________ |

| | | | |
|---|---|---|---|
| Data management | 19 | Plans for data entry, coding, security, and storage, including any related processes to promote data quality (eg, double data entry; range checks for data values). Reference to where details of data management procedures can be found, if not in the protocol | ______12______ |
| Statistical methods | 20a | Statistical methods for analysing primary and secondary outcomes. Reference to where other details of the statistical analysis plan can be found, if not in the protocol | ______12______ |
| | 20b | Methods for any additional analyses (eg, subgroup and adjusted analyses) | ______12______ |
| | 20c | Definition of analysis population relating to protocol non-adherence (eg, as randomised analysis), and any statistical methods to handle missing data (eg, multiple imputation) | ______12______ |
| **Methods: Monitoring** | | | |
| Data monitoring | 21a | Composition of data monitoring committee (DMC); summary of its role and reporting structure; statement of whether it is independent from the sponsor and competing interests; and reference to where further details about its charter can be found, if not in the protocol. Alternatively, an explanation of why a DMC is not needed | ______13______ |
| | 21b | Description of any interim analyses and stopping guidelines, including who will have access to these interim results and make the final decision to terminate the trial | ______14______ |
| Harms | 22 | Plans for collecting, assessing, reporting, and managing solicited and spontaneously reported adverse events and other unintended effects of trial interventions or trial conduct | ______________ |
| Auditing | 23 | Frequency and procedures for auditing trial conduct, if any, and whether the process will be independent from investigators and the sponsor | ______________ |
| **Ethics and dissemination** | | | |
| Research ethics approval | 24 | Plans for seeking research ethics committee/institutional review board (REC/IRB) approval | ______14______ |
| Protocol amendments | 25 | Plans for communicating important protocol modifications (eg, changes to eligibility criteria, outcomes, analyses) to relevant parties (eg, investigators, REC/IRBs, trial participants, trial registries, journals, regulators) | ______________ |


For peer review only - http://bmjopen.bmj.com/site/about/guidelines.xhtml

| | | | |
|---|---|---|---|
| Consent or assent | 26a | Who will obtain informed consent or assent from potential trial participants or authorised surrogates, and how (see Item 32) | ______14______ |
| | 26b | Additional consent provisions for collection and use of participant data and biological specimens in ancillary studies, if applicable | ______________ |
| Confidentiality | 27 | How personal information about potential and enrolled participants will be collected, shared, and maintained in order to protect confidentiality before, during, and after the trial | ______________ |
| Declaration of interests | 28 | Financial and other competing interests for principal investigators for the overall trial and each study site | ______14______ |
| Access to data | 29 | Statement of who will have access to the final trial dataset, and disclosure of contractual agreements that limit such access for investigators | ______12______ |
| Ancillary and post-trial care | 30 | Provisions, if any, for ancillary and post-trial care, and for compensation to those who suffer harm from trial participation | ______________ |
| Dissemination policy | 31a | Plans for investigators and sponsor to communicate trial results to participants, healthcare professionals, the public, and other relevant groups (eg, via publication, reporting in results databases, or other data sharing arrangements), including any publication restrictions | ______14______ |
| | 31b | Authorship eligibility guidelines and any intended use of professional writers | ______________ |
| | 31c | Plans, if any, for granting public access to the full protocol, participant-level dataset, and statistical code | ______________ |
| **Appendices** | | | |
| Informed consent materials | 32 | Model consent form and other related documentation given to participants and authorised surrogates | ______18______ |
| Biological specimens | 33 | Plans for collection, laboratory evaluation, and storage of biological specimens for genetic or molecular analysis in the current trial and for future use in ancillary studies, if applicable | ______________ |

*It is strongly recommended that this checklist be read in conjunction with the SPIRIT 2013 Explanation & Elaboration for important clarification on the items. Amendments to the protocol should be tracked and dated. The SPIRIT checklist is copyrighted by the SPIRIT Group under the Creative Commons "Attribution-NonCommercial-NoDerivs 3.0 Unported" license.

BMJ Open

# Study protocol: The EPOS-trial - The effect of air-filtration through a plasma chamber on the incidence of surgical site infection in orthopaedic surgery. A randomized, double-blind, placebo-controlled trial

| | |
|---|---|
| Journal: | *BMJ Open* |
| Manuscript ID | bmjopen-2020-047500.R2 |
| Article Type: | Protocol |
| Date Submitted by the Author: | 21-Dec-2021 |
| Complete List of Authors: | Persson, Anders; Karolinska Institutet Institutionen för kliniska vetenskaper Danderyds sjukhus,<br>Atroshi, Isam; Lund University, Department of Clinical Sciences; Hässleholm-Kristianstad Hospitals, Department of Orthopaedics<br>Tyszkiewicz, Thomas; Lund University, Department of Clinical Sciences; Hässleholm-Kristianstad Hospitals, Department of Orthopaedics<br>Hailer, Nils; Uppsala University, Department of Surgical Sciences; Uppsala University Hospital, Department of Orthopaedics<br>Lazarinis, Stergios; Uppsala University, Department of Surgical Sciences; Uppsala University Hospital, Department of Orthopaedics<br>Eisler, Thomas; Karolinska Institutet Institutionen för kliniska vetenskaper Danderyds sjukhus<br>Brismar, Harald; Karolinska Institute Department of Clinical Science Intervention and Technology; Karolinska Universitetsjukhuset i Huddinge, Department of Orthopaedics and Biotechnology<br>Mukka, Sebastian; Umea University, Department of Surgical and Perioperative Sciences; Umeå University Hospital<br>Kernell, Per-Juan; Löwenströmska Hospital, GHP Ortho Center Stockholm<br>Mohaddes, Maziar; Swedish Hip Arthroplasty Register; Institute of Clinical Sciences. Sahlgrenska Academy. University Of Gothenburg, Department of Orthopaedics<br>Sköldenberg, Olof; Karolinska Institutet Institutionen för kliniska vetenskaper Danderyds sjukhus<br>Gordon, Max; Karolinska Institutet Institutionen för kliniska vetenskaper Danderyds sjukhus |
| <b>Primary Subject Heading</b>: | Infectious diseases |
| Secondary Subject Heading: | Surgery |
| Keywords: | Orthopaedic & trauma surgery < SURGERY, Infection control < INFECTIOUS DISEASES, Hip < ORTHOPAEDIC & TRAUMA SURGERY, Adult orthopaedics < ORTHOPAEDIC & TRAUMA SURGERY, Knee < ORTHOPAEDIC & TRAUMA SURGERY |
| | |

# Study protocol: The EPOS-trial - The effect of air-filtration through a plasma chamber on the incidence of surgical site infection in orthopaedic surgery. A randomized, double-blind, placebo-controlled trial

Anders Persson[1], Isam Atroshi[2], Thomas Tyszkiewicz[2], Nils Hailer[3], Stergios Lazarinis[3], Thomas Eisler[1], Harald Brismar[4], Sebastian Mukka[5], Per-Juan Kernell[6], Maziar Mohaddes[7], Olof Sköldenberg[1], Max Gordon[1]

1. Karolinska Institutet, Department of Clinical Sciences, Danderyd Hospital, Division of Orthopaedics, Stockholm, Sweden
2. Lund University, Department of Clinical Sciences, Orthopaedics, Lund, and Department of Orthopaedics, Hässleholm-Kristianstad Hospitals, Kristianstad, Sweden
3. Uppsala University, Department of Surgical Sciences, Orthopaedics, Uppsala University Hospital, Uppsala, Sweden
4. Karolinska Institutet, Department of Clinical Science, Intervention and Technology, Department of Orthopaedics and Biotechnology, Karolinska University Hospital in Huddinge, Stockholm, Sweden
5. Umeå University, Department of Surgical and Perioperative Sciences, Umeå University Hospital, Umeå, Sweden
6. GHP Ortho Center Stockholm, Löwenströmska Hospital, Stockholm, Sweden
7. Department of Orthopaedics, Institute of Clinical Sciences, Sahlgrenska Academy, Gothenburg University, Gothenburg, Sweden, and The Swedish Arthroplasty Register, Gothenburg, Sweden

Correspondance: Anders Persson, M.D., PhD student, Department of Clinical Sciences, Dep. of Orthopaedics, Karolinska Institutet at Danderyd Hospital, S-18288 Danderyd, Sweden, tel: +46 8 12355000

E-mail: anders.persson@regionstockholm.se

**Key words:** surgical site infection, prevention, RCT, air-filtration, operating room, environment

**Word count:** 3553

For peer review only

## Abstract

### Introduction

There is controversy regarding the importance of air-transmitted infections for surgical site infections (SSI's) after orthopaedic surgery. Research has been hindered by both the inability in blinding the exposure, and by the need for recruiting large enough cohorts. The aim of this study is to investigate whether using a new form of air-purifier using plasma-air-purification (PAP) in operating rooms (OR's) lower the SSI-rate, or not.

### Methods and analysis

Multicenter, double-blind, cluster-randomized, placebo-controlled trial conducted at seven hospitals 2017-2022. All patients that undergo orthopaedic surgery for minimum 30 minutes are included. Intervention group: patients operated in OR with PAP-devices turned ON. Control group: patients operated in OR with PAP-devices turned OFF. Randomization: Each OR will be randomized in periods of 4, 6, or 8 weeks to either have the devices on or off. Primary outcome: Any SSI postoperatively defined as a composite endpoint of any of the following: use of isoxazolylpenicillin, clindamycin or rifampicin for 2 days or more, ICD codes or Nordic Medico-Statistical Committee (NOMESCO) codes indicating postoperative infection. In a second step, we will perform a chart review on those patients with positive indicators of SSI to further validate the outcome. Secondary outcomes are described in the methods' section. Power: We assume an SSI rate of 2%, an SSI reduction rate of 25% and we need approx. 45 000 patients to attain a power of 80% at a significance level of 0.05.

### Ethics and dissemination

The study is approved by the Swedish Ethical Review Authority. The interim analysis results from the study will be presented only to the researchers involved unless the study thereafter is interrupted for whatever reason. Publication in a medical journal will be presented after inclusion of the last patient.

### Registration details

The EPOS trial is registered at ClinicalTrials.gov with identifier NCT02695368.


For peer review only

## Strengths and limitations of this study

- ✓ EPOS is a multicenter, placebo-controlled trial with approximately 45 000 study subjects, that will evaluate the effect of plasma-air-purification on the incidence of surgical site infections after orthpaedic surgery.
- ✓ The double-blinded design provides strong internal validity to the study results.
- ✓ The cluster-randomization design, which is created through switching of the exposure within each operating room, minimizes the risk of allocation bias.
- ✓ This study is the first RCT to our knowlegde, investigating the true cause-and effect relationship between an air-purifying intervention in operating rooms and surgical site infections.
- ✓ The primary limitation to the study is the resource intensity, mainly due to the large number of study subjects required to study such an unusual outcome, and the concomitant review of medical records to validate the outcome.

For peer review only

# Introduction

Despite surgery in clean OR's, surface sterilization, and antibiotics, SSI after orthopaedic surgery have an overall estimated incidence of 1-4% (1-3). This feared complication is associated with long-term antibiotics, repeated surgeries, prolonged hospital stays, economic burden, and a poorer end result for individual patients (4, 5). Prevention of SSI's is therefore of paramount importance.

Air flow within the OR can spread airborne particles, posing a potential risk for postoperative infection. These airborne particles include dust, textile fibres, skin scales, and respiratory aerosols, loaded with viable microorganisms (including *Staphylococcus aureus*) having been released mainly from the surgical team members and patient into the surrounding air of the OR. These particles have been shown to settle onto surfaces including the surgical wound and instruments (6). Thus, air-transmitted infections is one of the main reasons for SSI (6), making this an interesting intervention target.

Ever since the ground-breaking study by Charnley in 1969 (7), which showed that cleaner air in the OR drastically improved infection rates after total hip arthroplasty, vast efforts have been made to address this issue. Lidwell carried on his work, showing that ultra-clean air (i.e. <10 colony-forming units (CFU)/$m^3$) in combination with body-exhaust suits and prophylactic antibiotics further improved infection rates (8-10). Based on their work, in combination with several reports showing that laminar air flow (LAF) reduces bacterial contamination in the OR air (11-13), many OR's are today equipped with LAF ventilation. Unfortunately though, when evaluated in large cohort studies, systematic reviews and meta-analyses, LAF systems in modern state-of-the-art OR's have so far failed to prove efficient in preventing infections, compared to conventional ventilation (14-19).

The plasma air-purification (PAP) system used in our current study is an air purifier that sterilizes the air particles through a plasma chamber (Figure 1). Air in the OR is pumped through the chamber, and by using a small current it transforms the air in the vicinity of the electrode into plasma, which eradicates any bacteria that pass through (Figure 2). The small size of the machine allows it to fit into any operating theatre without interfering with existing equipment (20).

Similar systems exists that use ultraviolet light to sterilize air particles and reduce the rate of hospital acquired infections (21, 22). There are though few peer-reviewed articles regarding air-purification. While we have not found any randomized clinical trials, there are randomised field trials, outside hospital settings, that have shown positive effects in vivo. In a blinded randomized field trial on healthy volunteers using air purifiers, a significant reduction in air-particles were seen and this also led to a reduction of stress hormone for the participants. (23)

In hospital and OR settings the PAP technology alone significantly reduces the number of colony-forming units (CFUs) of staphylococci (the most common infecting microorganism in SSI) from 49-97% (24). In non-randomized studies it has been shown to reduce respiratory infections, personnel sick-leave, and severe infectious outbreaks (22, 24). These effects have though not been validated in randomized clinical trials in operating room settings. However, this is also true for all methods of reducing air-borne pathogens in operating rooms


For peer review only - http://bmjopen.bmj.com/site/about/guidelines.xhtml

that currently are used, such as surgery in LAF OR's. The need for Level 1 evidence in this field of medicine is urgent.

The EPOS-trial is therefore designed as a multicentre, double-blinded, cluster-randomized, placebo-controlled trial.

The aim of this study is to investigate whether using PAP devices that clean the air from contaminating particles in OR's lower the rate of SSI, or not. The primary endpoint will be SSI rate within 12 weeks postoperatively, defined as either use of antibiotics targeting common implant pathogens, ICD code or NOMESCO code indicating postoperative infection. This proxy variable is more closely described in the methods section. We hypothesize that PAP can reduce the incidence of SSI in orthopaedic surgery by 25%. Secondary aims include investigating the number of prescribed antibiotics as well as the number of needed readmissions and length-of-stay for SSI.

For peer review only


For peer review only - http://bmjopen.bmj.com/site/about/guidelines.xhtml

# Methods: Participants, interventions and outcomes

### *Study setting*

The study is being conducted at seven major hospitals in Sweden (Table 2). Inclusion will take place between April 2017 and 31st of Dec 2021. The randomized clinical trial setting has been chosen to control for the huge number of possible confounders influencing the outcome. The study protocol has been written according to the SPIRIT (Standard Protocol Items for Randomized Trials) statement. A CONSORT flow diagram, published as a supplementary file to this protocol, describes our study graphically.

### *Eligibility criteria*

We will **include** (1) all patients that undergo surgery for 30 minutes or longer at each centre during the study period. We assume that surgeries lasting less than 30 minutes are less susceptible to SSI's. Including those in this study would therefore result in a larger cohort. We will **exclude** surgeries on 1) already infected surgical sites, defined as: ICD- or NOMESCO codes indicating infection (same as those used for the primary outcome, see below), open fractures, traumatic wounds, vacuum assisted wound therapy, 2) patients that have withdrawn antibiotics 2 weeks or less prior to surgery, and 3) patients that have actively marked their hospital charts with an added privacy notice. If patients have multiple surgeries during the study period, only the first operation will be included.

### *Intervention*

At each hospital, all OR's that perform surgery on orthopaedic patients will be equipped with three (3) PAP-systems each. The groups are defined as: **Intervention group:** those operated where the PAP device has been turned on for at least 2 days prior to index surgery. **Control group:** those where the PAP device has been turned off for at least 2 days prior to index surgery. **Mixed group:** those receiving surgeries in OR's within 2 days after the PAP device switches status. We will also in the analysis sub-group the study subjects according to measurements prior to study start into: ***Regular*** operating rooms (≥10 CFU/ $m^3$) and ***Ultra-clean*** operating rooms (<10 CFU/$m^3$).

PAP device status and function will be monitored continuously during the inclusion period through standardized manual controls every 3 months, and also at the end of the inclusion period by validating PAP device status retrospectively through memory card recordings in each device.

### *Outcomes*

The **primary outcome** is any indication of SSI within 12 weeks postoperatively, defined as a composite endpoint of any of the following:

1. Withdrawal or other documented use of antibiotics corresponding to 2 days or more after surgery targeting Staphylococcus aureus
2. ICD code indicating postoperative infection (at date of readmission)
3. NOMESCO code indicating postoperative infection


For peer review only - http://bmjopen.bmj.com/site/about/guidelines.xhtml

In a second step, we will perform a chart review on those patients with positive indicators of SSI to further validate the outcome. We will use the internationally accepted CDC definition of SSI when finally establishing that a SSI has occurred (Table 1) (25).

The **effect size** of the primary endpoint is the relative risk of contracting SSI for the intervention group versus the control group and will be calculated by dividing the probability of contracting a SSI in the intervention group by the control group = $\frac{SSI\ rate\ intervention\ group}{SSI\ rate\ control\ group}$.
The **effect size** will also be presented as an absolute risk difference = $SSI\ rate\ intervention\ group - SSI\ rate\ control\ group$.

The primary outcome is a **surrogate variable** for SSI as it, due to the large sample size is practically impossible to have all patients come back for outpatient visits and be visually inspected. To further validate the outcome, a medical record review will be performed in a second step on all individual patients with indication of SSI. Our expectations are that the choice and succeeding validation of this proxy variable can provide us with a reliable tool for investigating SSI's in future projects.

The Swedish health-care registers, especially the Prescribed Drug Register and the Patient Register will make sure that we get an almost complete (>99%) coverage of all relevant SSI's.

The **secondary outcomes** are:

a) Withdrawal or other documented use of any antibiotics for 2 days or more after surgery during the first 30 postoperative days
b) Number of days with antibiotics during the first 30 days
c) Same as a) & b) but up to 90 days after surgery
d) Death during the first 2 postoperative years

These analyses will also be performed with and without adjustment for pre-operative antibiotic use 6 months prior to the surgery.

### *Sample size*

The re-operation rate in Sweden due to infections within the first 2 years after surgery is 1.3% (3) for primary total hip replacements (THR's). This does not include THR's in fracture patients and other types of surgeries that are more susceptible to infections. We therefore assume that the SSI rate in our study population is 2% (1-3). Similarly, we know from other data that about 0.7% withdraw the antibiotics associated with the primary outcome within a 3 months' period prior to surgery. To account for infections unrelated to surgery we assume that the infection noise rate, i.e. non-surgical site infections, is less than 2%.
***Multicentre power with ultra-clean air:*** Hospitals with OR's with ultra-clean air may be less susceptible to the effect with an already lower infection rate. Pre-study CFU measurements suggest that approximately 80% of the included OR's are ultra-clean. If we assume that the infection rate is 2% or less, and that the effect size is reduced to 25% in an ultra-clean environment, we will need to recruit 22,630 patients in each group, i.e. approximately 45,000 patients to attain a power of 80% with a significance level of 0.05. This will be our target population. We expect a very low drop-out rate/missing data in the study, estimated to be <1%. At the interim analysis the p-value will be set 10 times lower; at 0.005; i.e. if a statistically significant result between the groups is observed at 18 months, the study will be


For peer review only - http://bmjopen.bmj.com/site/about/guidelines.xhtml

stopped. See Figure 3 and Appendix 1 for details in R and explanation of the script for the sample size analysis.

### *Recruitment*

We estimate that with only one center, such as Danderyd Hospital, recruitment would take >5 years. The hospital operates about 4,500 patients each year and as it is unlikely that more than 500 of these will be excluded. We therefore anticipate recruiting 4,000 patients/year. Performing this study in a multi-center setting is therefore crucial. In Table 2 the participating centers are presented, and their estimated proportion of patients included.

### *Patient and Public Involvement*

Representatives of the Swedish Osteoporosis Society (www.osteoporos.org) and the Swedish Rheumatic Society (www.reumatiker.se) are members of the study steering committee, and participate mainly in discussions regarding plans for reporting and publishing the results of the study.

For peer review only



For peer review only - http://bmjopen.bmj.com/site/about/guidelines.xhtml

## Methods: Assignment of interventions

### *Allocation*

The three PAP devices in each OR will synchronically be randomized in periods of 4, 6, or 8 weeks to either have the system “on” or “off”. The switch will always occur midnight Friday in order to limit the patients exposed to partial effect during the first two days after switching status. The system can be programmed to be active (i.e. plasma chamber eradicating bacteria) at any given timeframe. The manufacturer of the PAP devices will prepare the randomization allocation and automatic execution of it. The randomization sequence is at minimum 8 years long and will be submitted to a third, independent party, responsible for keeping the allocation secret until interim analyses or study end. At the interim analyses only allocations up to that date will be released.

### *Blinding*

The on/off only refers to the plasma chamber responsible for the antimicrobial effect. As the machine retains the air flow it will be impossible for staff, surgeons and patients to determine the status of the machine from the outside. The device will also automatically switch status where the true status is concealed for all study participants including other hospital personnel until the end of the study.

For peer review only



For peer review only - http://bmjopen.bmj.com/site/about/guidelines.xhtml

# Methods: Data collection, management, and analysis

## *Data collection methods*

For the primary endpoint the following codes will be used to detect if individual patients have contracted an SSI following surgery. If any of these codes indicate SSI, a chart review will be performed in a second step to verify the outcome:

1. From The Swedish Prescribed Drug Register: withdrawal of antibiotics targeting Staphylococcus aureus corresponding to a minimum amount of 2 defined daily dosages (an estimate provided by the registry for the expected daily dosage). The date of withdrawal will serve as an indicator of treatment start unless inpatient data is available with more granular information. The drug ATC codes considered to target relevant bacteria are:
    a. J01CF05 (isoxazolylpenicillin)
    b. J01FF01 (clindamycin)
    c. J04AB02 (rifampicin)
2. From The National Patient Registry: ICD trigger codes indicating postoperative infections:
    a. T793 – Post-traumatic wound infection, not elsewhere classified
    b. T814 – Infection following a procedure, not elsewhere classified
    c. T84[5-7] – Infection and inflammatory reaction due to internal joint prosthesis, internal fixation device, or other internal orthopaedic prosthetic devices, implants and grafts
    d. T874 – Infection of amputation stump
    e. B9[5,6,8] – Bacterial specification
    f. L0[2-4] – Cutaneous abscess, furuncle and carbuncle; cellulitis
    g. A[24]6 – Erysipeloid, erysipelas
3. From The National Patient Registry: NOMESCO trigger codes indicating postoperative infections:
    a. Incision abscess: TN[A-H]05
    b. Surgeries due infections: N[A-H]S[0-4,9]9
    c. Extremities wound revision: Q[CD]B05
    d. Re-operation for infection: N[A-H]W69
    e. Vacuum treatment: DQ023

Both local and national registry data will be used according to availability. For the admission episodes with the code indicators the admission date is the index date, i.e. if an admission occurs after 91 days with a trigger code it will not be considered an indicator of a postoperative infection.

In-hospital information systems will supply information on:

a) Patient ID
b) Date(s) of surgery
c) Surgery associated codes including operated side
d) Operating room
e) In-hospital antibiotics

Both the surgical and medical data records will be retrieved depending on availability. Only centres that can provide the above data will be allowed to participate.



For peer review only - http://bmjopen.bmj.com/site/about/guidelines.xhtml

The Swedish National Patient Register includes all in-patient care and outpatient visits in Sweden with discharge codes according to ICD-10, NOMESCO codes and admission/discharge dates (26).

The Swedish Prescribed Drug Register (PDR) includes any withdrawn prescriptions. Prescriptions that are never withdrawn by patients and drugs bought over the counter without prescriptions are not included. The data fields used were the drug ATC-code, number of pills, and prescription text (27).

### *Data management*

The study data will be securely managed and stored encrypted at a computer within Karolinska Institutet at Danderyd Hospital. No other than the authors stated above will gain access to raw data.

### *Statistical methods*

The primary outcome is a binary variable where there are three groups. We will use logistic regression where the reference group is the placebo group, and the significance is related to the intervention group. The estimate for the mixed group is only for relating dose-effect, i.e. the group will not be pooled with either the placebo or the intervention group. Due to the randomization we do not intend to have any other covariates as confounders in the model. Similar methodology will be applied to the antibiotic's binary outcome. The number of days with antibiotics will be modelled using a linear regression with the similar interpretation to above regarding predictors. Mortality will be modelled using a cox proportional hazards model with time since surgery calculated as time to death, migration or 2 years. The cox model will not contain any covariates due to the randomized study nature. The analysis will be performed by an epidemiologist/statistician in our team (MG) and will be performed as a per-protocol analysis.

We will handle confounding by including only the patient's 1st surgical procedure. The randomization process will handle other confounding such as confounding by selection. Procedural confounding will be handled by the external part who has done the randomization procedure for each PAP device. Regarding dropouts the health-care registers and chart review done in the study will ensure a very low (<1%) drop-out rate. Individual patients can also request that they are excluded from the registers and thus from the study, but by experience, this is very rare and will also not affect our ability to analyse our primary and secondary endpoints.



For peer review only - http://bmjopen.bmj.com/site/about/guidelines.xhtml

## Methods: Monitoring

### *Data monitoring*

At 12 months after study start (2018-04-17) an interim analysis will be performed and the recruitment rate from each center will be evaluated. The study recruitment will end once we have reached a minimum of 45,000 patients. During the 2nd half of 2017/early 2018, the data quality of each recruiting center will be evaluated by extracting data from each center's hospital information center.

## Expected results

Our hypothesis is that the usage of this air-purifier significantly lowers the incidence of surgical site infections after orthopaedic surgery. Since the installation, management and purchase of this kind of machines is nowhere close to the resource intensity of other types of OR ventilation arrangements, it has the possibility of introducing a cost-effective instrument to prevent postoperative infections. Furthermore, this would perhaps benefit especially resource-scarce communities globally.

Secondarily, the large amount of data derived from this study, can subsequently be used analysing the effect of other kinds of exposures on the incidence of postoperative infections.

For peer review only

## Ethics and dissemination

The study is being conducted in accordance with the ethical principles of the Helsinki Declaration, and is approved by the Swedish Ethical Review Authority (2015/1139-31/4).

The interim analysis results from the study will be presented only to the researchers involved unless the study thereafter is interrupted due to significant difference in infection rate between the two groups. Publication in a medical journal will be presented after inclusion of the last patient.

Study participant information will be published on the hospital web site (see Appendix 2). Due to feasibility reasons in a study with approximately 45 000 study participants, and the very low probability of any adverse effects related to the intervention, no personal consent forms will be collected. However, individual patients can request exclusion from the data analysis.

The EPOS trial is registered at ClinicalTrials.gov with identifier NCT02695368.

## Author contributions

AP operates the trial, and led the writing of this manuscript, with contributions from the rest of the authors. TE, MM, HB, NH, SL, TT, IA, SM and PK operate the inclusion centers. MG and OS designed the original study and developed the protocol. MG is the responsible statistician and supervises the study. All authors contributed to the editing and redrafting of the manuscript.

## Funding statement

8.8 million SEK from the Swedish Research Council has been received for 2017-2020 (grant number 2017-00198). 1.4 million SEK from ALF (Stockholm County and Karolinska Institute, application number 20160251, record number LS 2015-1198) has been received for 2017-2018. We have a discounted price for the rental of the machines but have chosen not to apply for funding from the manufacturers of the plasma-air-purifier equipment to ensure that the study is independent.

## Competing interests statement

None.

For peer review only

## References

1. Reilly J, Allardice G, Bruce J, Hill R, McCoubrey J. Procedure-specific surgical site infection rates and postdischarge surveillance in Scotland. Infection control and hospital epidemiology. 2006;27(12):1318-23.
2. Pull ter Gunne AF, Cohen DB. Incidence, prevalence, and analysis of risk factors for surgical site infection following adult spinal surgery. Spine. 2009;34(13):1422-8.
3. Lindgren JV, Gordon M, Wretenberg P, Kärrholm J, Garellick G. Validation of reoperations due to infection in the Swedish Hip Arthroplasty Register. BMC musculoskeletal disorders. 2014;15:384.
4. de Lissovoy G, Fraeman K, Hutchins V, Murphy D, Song D, Vaughn BB. Surgical site infection: incidence and impact on hospital utilization and treatment costs. Am J Infect Control. 2009;37(5):387-97.
5. Bozic KJ, Katz P, Cisternas M, Ono L, Ries MD, Showstack J. Hospital resource utilization for primary and revision total hip arthroplasty. J Bone Joint Surg Am. 2005;87(3):570-6.
6. Fernstrom A, Goldblatt M. Aerobiology and its role in the transmission of infectious diseases. Journal of pathogens. 2013;2013:493960.
7. Charnley J, Eftekhar N. Postoperative infection in total prosthetic replacement arthroplasty of the hip-joint. With special reference to the bacterial content of the air of the operating room. Br J Surg. 1969;56(9):641-9.
8. Lidwell OM, Elson RA, Lowbury EJ, Whyte W, Blowers R, Stanley SJ, et al. Ultraclean air and antibiotics for prevention of postoperative infection. A multicenter study of 8,052 joint replacement operations. Acta orthopaedica Scandinavica. 1987;58(1):4-13.
9. Lidwell OM, Lowbury EJ, Whyte W, Blowers R, Stanley SJ, Lowe D. Airborne contamination of wounds in joint replacement operations: the relationship to sepsis rates. J Hosp Infect. 1983;4(2):111-31.
10. Lidwell OM, Lowbury EJ, Whyte W, Blowers R, Stanley SJ, Lowe D. Effect of ultraclean air in operating rooms on deep sepsis in the joint after total hip or knee replacement: a randomised study. British medical journal (Clinical research ed). 1982;285(6334):10-4.
11. Hirsch T, Hubert H, Fischer S, Lahmer A, Lehnhardt M, Steinau HU, et al. Bacterial burden in the operating room: impact of airflow systems. Am J Infect Control. 2012;40(7):e228-32.
12. Diab-Elschahawi M, Berger J, Blacky A, Kimberger O, Oguz R, Kuelpmann R, et al. Impact of different-sized laminar air flow versus no laminar air flow on bacterial counts in the operating room during orthopedic surgery. Am J Infect Control. 2011;39(7):e25-9.
13. Romano F, Milani S, Ricci R, Joppolo CM. Operating Theatre Ventilation Systems and Their Performance in Contamination Control: "At Rest" and "In Operation" Particle and Microbial Measurements Made in an Italian Large and Multi-Year Inspection Campaign. Int J Environ Res Public Health. 2020;17(19).
14. WHO. Global Guidelines for the Prevention of Surgical Site Infection. WHO Guidelines Approved by the Guidelines Review Committee. Geneva2016.
15. Breier AC, Brandt C, Sohr D, Geffers C, Gastmeier P. Laminar airflow ceiling size: no impact on infection rates following hip and knee prosthesis. Infection control and hospital epidemiology. 2011;32(11):1097-102.
16. Gastmeier P, Breier AC, Brandt C. Influence of laminar airflow on prosthetic joint infections: a systematic review. J Hosp Infect. 2012;81(2):73-8.
17. Bischoff P, Kubilay NZ, Allegranzi B, Egger M, Gastmeier P. Effect of laminar airflow ventilation on surgical site infections: a systematic review and meta-analysis. Lancet Infect Dis. 2017;17(5):553-61.
18. McHugh SM, Hill AD, Humphreys H. Laminar airflow and the prevention of surgical site infection. More harm than good? Surgeon. 2015;13(1):52-8.
19. Pasquarella C, Barchitta M, D'Alessandro D, Cristina ML, Mura I, Nobile M, et al. Heating, ventilation and air conditioning (HVAC) system, microbial air contamination and


For peer review only - http://bmjopen.bmj.com/site/about/guidelines.xhtml

surgical site infection in hip and knee arthroplasties: the GISIO-SItI Ischia study. Ann Ig. 2018;30(5 Supple 2):22-35.
20. Novaerus Technology 2020 [Available from: https://cmsifyassets-1290.kxcdn.com/nov-production/uploads/asset/1741/attachment/NV_NanoStrike_Brochure_HIRES.pdf.
21. Anis HK, Curtis GL, Klika AK, Piuzzi NS, Otiso J, Richter SS, et al. In-Room Ultraviolet Air Filtration Units Reduce Airborne Particles During Total Joint Arthroplasty. J Orthop Res. 2020;38(2):431-7.
22. Kane D. Study shows reduced HAIs, airborne pathogens with in-room active UV-C system http://www.mcknights.com/marketplace/study-shows-reduced-hais-airborne-pathogens-with-in-room-active-uv-c-system/article/573958/2017 [
23. Li H, Cai J, Chen R, Zhao Z, Ying Z, Wang L, et al. Particulate Matter Exposure and Stress Hormone Levels: A Randomized, Double-Blind, Crossover Trial of Air Purification. Circulation. 2017;136(7):618-27.
24. James E. Independent validation report for Novareus air treatment systems 2014 [Available from: http://sandbox.novaerus.com/wp-content/uploads/2014/03/Independent-Validation-Report-for-Novaerus-NHS.pdf.
25. Horan TC, Gaynes RP, Martone WJ, Jarvis WR, Emori TG. CDC definitions of nosocomial surgical site infections, 1992: a modification of CDC definitions of surgical wound infections. Infection control and hospital epidemiology. 1992;13(10):606-8.
26. The National Patient Register [Internet]. 2019. Available from: https://www.socialstyrelsen.se/en/statistics-and-data/registers/register-information/the-national-patient-register/.
27. Prescribed Drug Register [Internet]. 2019. Available from: https://www.socialstyrelsen.se/statistik-och-data/register/alla-register/lakemedelsregistret/.

For peer review only

# Tables

Table 1. CDC definition of surgical site infection (SSI)

**Superficial Incisional Surgical Site Infection**

Infection within 30 days after the operation and only involves skin and subcutaneous tissue of the incision and at least one of the following:

1. Purulent drainage with or without laboratory confirmation, from the superficial incision.
2. Organisms isolated from an aseptically obtained culture of fluid or tissue from the superficial incision.
3. At least one of the following signs or symptoms of infection: pain or tenderness, localised swelling, redness, or heat and superficial incision is deliberately opened by surgeon, unless incision is culture-negative.
4. Diagnosis of superficial incisional SSI made by a surgeon or attending physician.

**Deep Incisional Surgical Site Infection**

Infection occurs within 30 days after the operation if no implant is left in place or within one year if implant is in place and the infection appears to be related to the operation and infection involves deep soft tissue (e.g. fascia, muscle) of the incision and at least one of the following:

1. Purulent drainage from the deep incision but not from the organ/space component of the surgical site.
2. A deep incision spontaneously dehisces or is deliberately opened by a surgeon when the patient has at least one of the following signs or symptoms: fever (>38°C), localised pain or tenderness, unless incision is culture-negative.
3. An abscess or other evidence of infection involving the deep incision is found on direct examination, during reoperation, or by histopathologic or radiologic examination.
4. Diagnosis of deep incisional SSI made by a surgeon or attending physician.

**Organ/Space Surgical Site Infection**

Infection occurs within 30 days after the operation if no implant is left in place or within one year if implant is in place and the infection appears to be related to the operation and infection involves any part of the anatomy (e.g., organs and spaces) other than the incision which was opened or manipulated during an operation and at least one of the following:

1. Purulent drainage from a drain that is placed through a stab wound into the organ/space.
2. Organisms isolated from an aseptically obtained culture of fluid or tissue in the organ/space
3. An abscess or other evidence of infection involving the organ/space that is found on direct examination, during reoperation, or by histopathologic or radiologic examination.
4. Diagnosis of organ/space SSI made by a surgeon or attending physician.

Table 2. Recruitment centres and estimated recruitment based on number of surgeries performed annually. The hospitals are already recruiting patients.

| Centre | Estimated n | % recruited |
|---|---|---|
| Danderyd Hospital | 8000 | 18% |
| Hässleholm/Kristianstad | 10 000 | 22% |
| Huddinge hospital | 6000 | 13% |
| Akademiska hospital | 10 000 | 22% |
| Ortho Center | 3000 | 7% |
| Umeå | 8000 | 18% |
| **Total sample size** | **45000** | **100%** |


For peer review only - http://bmjopen.bmj.com/site/about/guidelines.xhtml

## Figures

1. Rendered view of Novaerus NV800
2. Air-flow through the air-purifier
3. Graphs showing the impact of base SSI rate and rate of unrelated infections on the required sample size

For peer review only

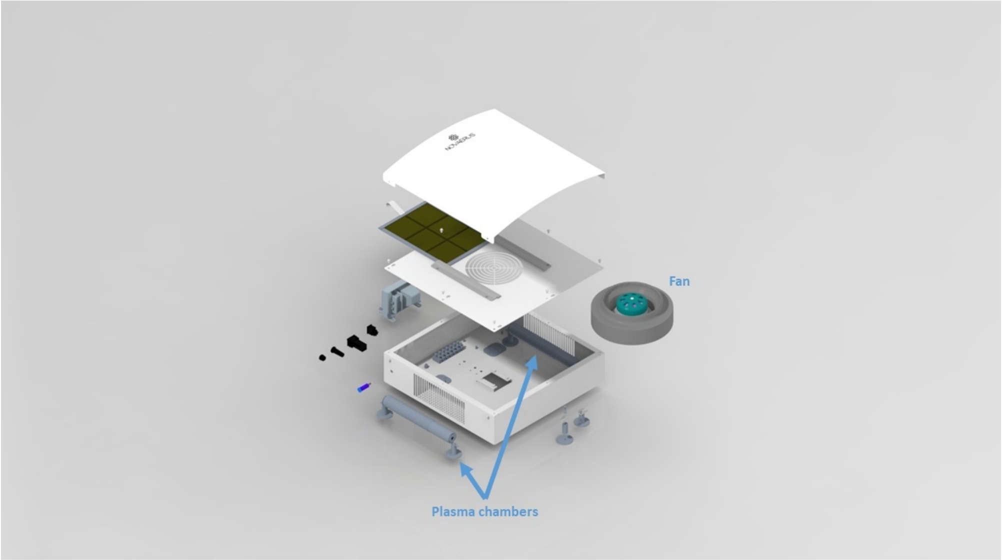


Rendered view of Novaerus NV800

338x190mm (96 x 96 DPI)

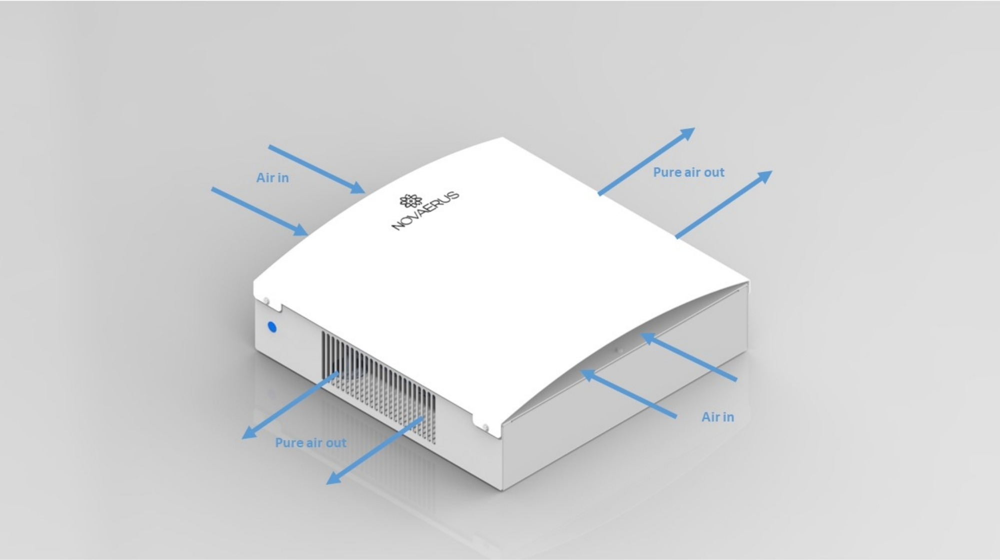


Air flow through the air-purifier

338x190mm (96 x 96 DPI)

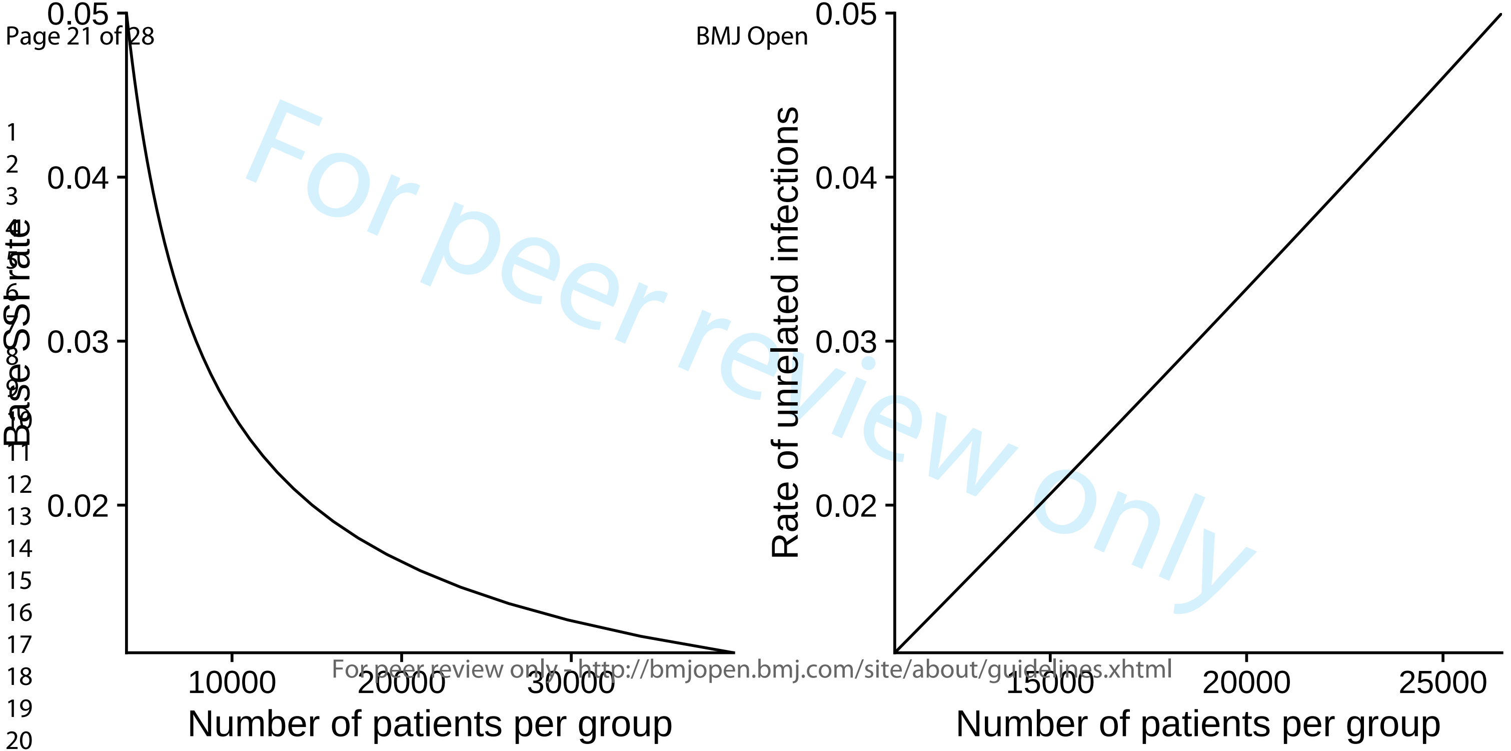


For peer review only

# Appendix 1

## *1 R-script power analysis*

```
# A certain number of infections in the dataset will not be due
# to the surgery that will be adding noise ot the calcualtion.
# We estimate this to 2% based on previous drug registry study experience
unrelated_rate <- 2/100
sig_lvl <- 0.05 # 0.05 with multiplicity for interim analysis of 0.005 added

##############################################
# Power calculation for all regular theaters #
##############################################
# The effect size in a regular operating theater
efx_size <- 0.4
# Base rate of SSI
inf.rate_base <- 2/100
# The SSI rate after the effect
inf.rate_efx <- inf.rate_base*(1-efx_size)
# The power is a combination of the unrelated rate + the rates of interest
power.prop.test(p1 = unrelated_rate + inf.rate_base,
                p2 = unrelated_rate + inf.rate_efx,
                sig.level = sig_lvl,
                power = 0.8)


##########################################
# Power calculation for a combination of #
# regular and ultra-clean theaters       #
##########################################
# The reduced effect in ultra-clean environments
efx_size_clean <- 0.25
# The expected infection rate in the ultraclean operating theaters
inf.rate_clean <- inf.rate_base * 0.8
# The proportion of ultra-clean operating theaters
prop_clean <- 0.8
# The SSI rate when combining the effect in regular and ultraclean operating theaters
inf.mix <- inf.rate_base * (1-prop_clean) +
  inf.rate_clean * prop_clean
# The reduced rate for above combination
inf.mix_efx <- inf.rate_base *
  (1-prop_clean) *
  (1-efx_size) +
  inf.rate_clean *
  prop_clean *
  (1-efx_size_clean)
# The power is a combination of the unrelated rate + the rates of interest
power.prop.test(p1 = unrelated_rate + inf.mix,
                p2 = unrelated_rate + inf.mix_efx,
                sig.level = sig_lvl,
                power = 0.8)
```

# Appendix 2

### *Informed consent material (freely translated from Swedish)*

Since December 2016, there is an ongoing randomized trial at the XXXX Hospital, in which all patients that undergo orthopaedic surgery are included. The trial is performed to evaluate a new technique for air-purification in the operating rooms. We will perform follow-ups of all patients treated with surgery in those operating rooms, to check if the new air-purification has reduced the infection rate. Due to the large number of included patients in the study (approx. 45 000), the only data compiled for analysis will be the one already collected for mandatory national registries. If you had surgery performed at our department during this period of time, and wish more information regarding this study, or if you wish not to be included in the analysis part of the study, please contact any of the persons listed below:

| | | |
|---|---|---|
| Anders Persson | Max Gordon | Olof Sköldenberg |
| e-mail: | e-mail: | e-mail: |
| tel: | tel: | tel: |
| role: | role: | role: |

For peer review only

CONSORT
TRANSPARENT REPORTING of TRIALS

## CONSORT Flow Diagram – Study plan for EPOS

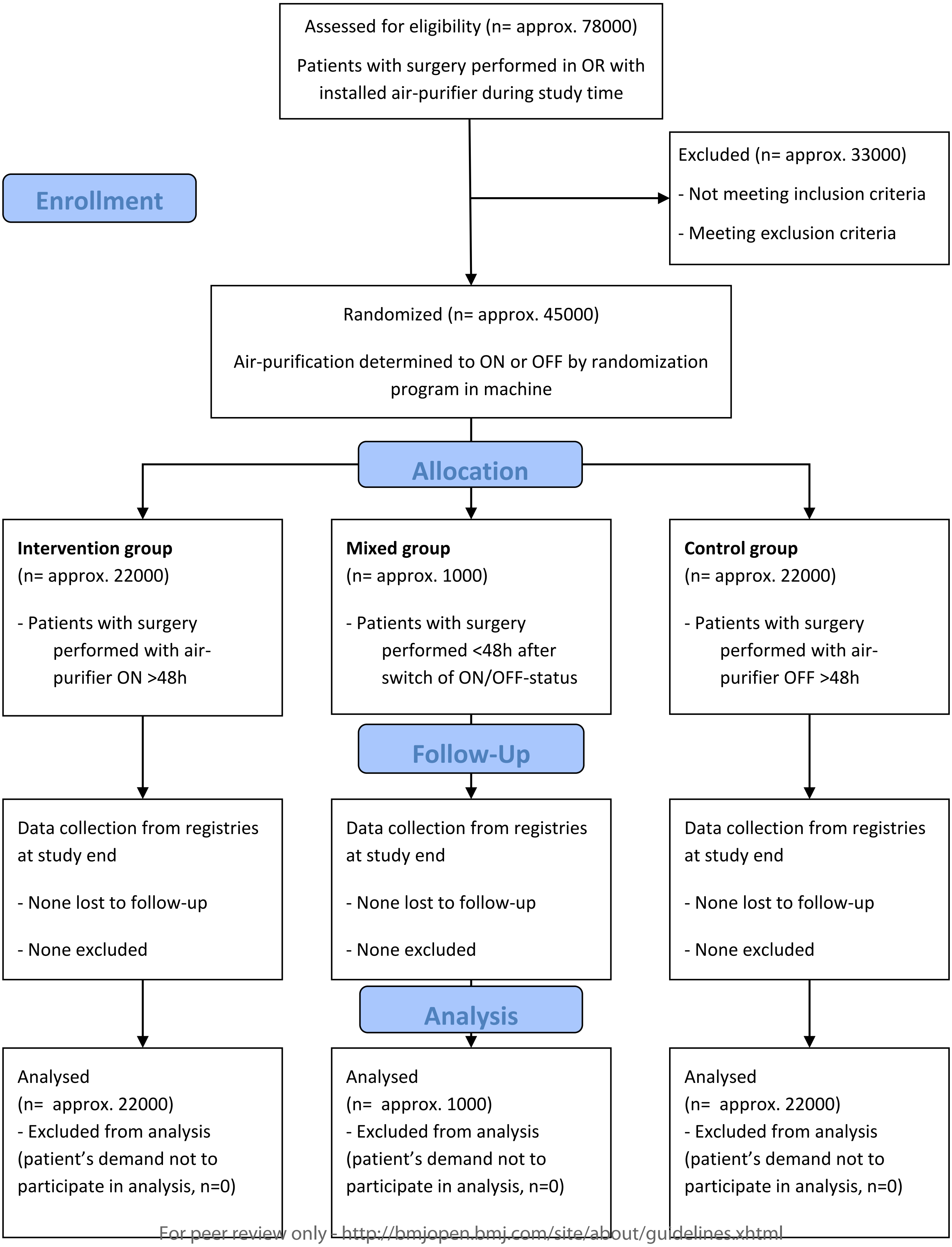


For peer review only - http://bmjopen.bmj.com/site/about/guidelines.xhtml

SPIRIT

STANDARD PROTOCOL ITEMS: RECOMMENDATIONS FOR INTERVENTIONAL TRIALS

SPIRIT 2013 Checklist: Recommended items to address in a clinical trial protocol and related documents*

| Section/item | Item No | Description | Addressed on page number |
|---|---|---|---|
| **Administrative information** | | | |
| Title | 1 | Descriptive title identifying the study design, population, interventions, and, if applicable, trial acronym | ______1________ |
| Trial registration | 2a | Trial identifier and registry name. If not yet registered, name of intended registry | ______2________ |
| | 2b | All items from the World Health Organization Trial Registration Data Set | ______________ |
| Protocol version | 3 | Date and version identifier | ______________ |
| Funding | 4 | Sources and types of financial, material, and other support | ______14_______ |
| Roles and responsibilities | 5a | Names, affiliations, and roles of protocol contributors | ______1, 14____ |
| | 5b | Name and contact information for the trial sponsor | ______1________ |
| | 5c | Role of study sponsor and funders, if any, in study design; collection, management, analysis, and interpretation of data; writing of the report; and the decision to submit the report for publication, including whether they will have ultimate authority over any of these activities | ______10, 14___ |
| | 5d | Composition, roles, and responsibilities of the coordinating centre, steering committee, endpoint adjudication committee, data management team, and other individuals or groups overseeing the trial, if applicable (see Item 21a for data monitoring committee) | ______________ |

For peer review only



For peer review only - http://bmjopen.bmj.com/site/about/guidelines.xhtml

**Introduction**

| | | | |
|---|---|---|---|
| Background and rationale | 6a | Description of research question and justification for undertaking the trial, including summary of relevant studies (published and unpublished) examining benefits and harms for each intervention | _____4_______ |
| | 6b | Explanation for choice of comparators | _____4_______ |
| Objectives | 7 | Specific objectives or hypotheses | _____5_______ |
| Trial design | 8 | Description of trial design including type of trial (eg, parallel group, crossover, factorial, single group), allocation ratio, and framework (eg, superiority, equivalence, noninferiority, exploratory) | _____5_______ |

**Methods: Participants, interventions, and outcomes**

| | | | |
|---|---|---|---|
| Study setting | 9 | Description of study settings (eg, community clinic, academic hospital) and list of countries where data will be collected. Reference to where list of study sites can be obtained | _____6_______ |
| Eligibility criteria | 10 | Inclusion and exclusion criteria for participants. If applicable, eligibility criteria for study centres and individuals who will perform the interventions (eg, surgeons, psychotherapists) | _____6_______ |
| Interventions | 11a | Interventions for each group with sufficient detail to allow replication, including how and when they will be administered | _____6_______ |
| | 11b | Criteria for discontinuing or modifying allocated interventions for a given trial participant (eg, drug dose change in response to harms, participant request, or improving/worsening disease) | ____________ |
| | 11c | Strategies to improve adherence to intervention protocols, and any procedures for monitoring adherence (eg, drug tablet return, laboratory tests) | _____6_______ |
| | 11d | Relevant concomitant care and interventions that are permitted or prohibited during the trial | ____________ |
| Outcomes | 12 | Primary, secondary, and other outcomes, including the specific measurement variable (eg, systolic blood pressure), analysis metric (eg, change from baseline, final value, time to event), method of aggregation (eg, median, proportion), and time point for each outcome. Explanation of the clinical relevance of chosen efficacy and harm outcomes is strongly recommended | _____6,7_____ |
| Participant timeline | 13 | Time schedule of enrolment, interventions (including any run-ins and washouts), assessments, and visits for participants. A schematic diagram is highly recommended (see Figure) | _____6,7_____ |


For peer review only - http://bmjopen.bmj.com/site/about/guidelines.xhtml

| | | | |
|---|---|---|---|
| Sample size | 14 | Estimated number of participants needed to achieve study objectives and how it was determined, including clinical and statistical assumptions supporting any sample size calculations | _____8_______ |
| Recruitment | 15 | Strategies for achieving adequate participant enrolment to reach target sample size | _____9_______ |

**Methods: Assignment of interventions (for controlled trials)**

Allocation:

| | | | |
|---|---|---|---|
| Sequence generation | 16a | Method of generating the allocation sequence (eg, computer-generated random numbers), and list of any factors for stratification. To reduce predictability of a random sequence, details of any planned restriction (eg, blocking) should be provided in a separate document that is unavailable to those who enrol participants or assign interventions | _____10______ |
| Allocation concealment mechanism | 16b | Mechanism of implementing the allocation sequence (eg, central telephone; sequentially numbered, opaque, sealed envelopes), describing any steps to conceal the sequence until interventions are assigned | _____10______ |
| Implementation | 16c | Who will generate the allocation sequence, who will enrol participants, and who will assign participants to interventions | _____10______ |
| Blinding (masking) | 17a | Who will be blinded after assignment to interventions (eg, trial participants, care providers, outcome assessors, data analysts), and how | _____10______ |
| | 17b | If blinded, circumstances under which unblinding is permissible, and procedure for revealing a participant's allocated intervention during the trial | _____________ |

**Methods: Data collection, management, and analysis**

| | | | |
|---|---|---|---|
| Data collection methods | 18a | Plans for assessment and collection of outcome, baseline, and other trial data, including any related processes to promote data quality (eg, duplicate measurements, training of assessors) and a description of study instruments (eg, questionnaires, laboratory tests) along with their reliability and validity, if known. Reference to where data collection forms can be found, if not in the protocol | _____11______ |
| | 18b | Plans to promote participant retention and complete follow-up, including list of any outcome data to be collected for participants who discontinue or deviate from intervention protocols | _____________ |

| | | | |
|---|---|---|---|
| Data management | 19 | Plans for data entry, coding, security, and storage, including any related processes to promote data quality (eg, double data entry; range checks for data values). Reference to where details of data management procedures can be found, if not in the protocol | ______12______ |
| Statistical methods | 20a | Statistical methods for analysing primary and secondary outcomes. Reference to where other details of the statistical analysis plan can be found, if not in the protocol | ______12______ |
| | 20b | Methods for any additional analyses (eg, subgroup and adjusted analyses) | ______12______ |
| | 20c | Definition of analysis population relating to protocol non-adherence (eg, as randomised analysis), and any statistical methods to handle missing data (eg, multiple imputation) | ______12______ |
| **Methods: Monitoring** | | | |
| Data monitoring | 21a | Composition of data monitoring committee (DMC); summary of its role and reporting structure; statement of whether it is independent from the sponsor and competing interests; and reference to where further details about its charter can be found, if not in the protocol. Alternatively, an explanation of why a DMC is not needed | ______13______ |
| | 21b | Description of any interim analyses and stopping guidelines, including who will have access to these interim results and make the final decision to terminate the trial | ______14______ |
| Harms | 22 | Plans for collecting, assessing, reporting, and managing solicited and spontaneously reported adverse events and other unintended effects of trial interventions or trial conduct | ____________ |
| Auditing | 23 | Frequency and procedures for auditing trial conduct, if any, and whether the process will be independent from investigators and the sponsor | ____________ |
| **Ethics and dissemination** | | | |
| Research ethics approval | 24 | Plans for seeking research ethics committee/institutional review board (REC/IRB) approval | ______14______ |
| Protocol amendments | 25 | Plans for communicating important protocol modifications (eg, changes to eligibility criteria, outcomes, analyses) to relevant parties (eg, investigators, REC/IRBs, trial participants, trial registries, journals, regulators) | ____________ |

| | | | |
|---|---|---|---|
| Consent or assent | 26a | Who will obtain informed consent or assent from potential trial participants or authorised surrogates, and how (see Item 32) | ______14______ |
| | 26b | Additional consent provisions for collection and use of participant data and biological specimens in ancillary studies, if applicable | ______________ |
| Confidentiality | 27 | How personal information about potential and enrolled participants will be collected, shared, and maintained in order to protect confidentiality before, during, and after the trial | ______________ |
| Declaration of interests | 28 | Financial and other competing interests for principal investigators for the overall trial and each study site | ______14______ |
| Access to data | 29 | Statement of who will have access to the final trial dataset, and disclosure of contractual agreements that limit such access for investigators | ______12______ |
| Ancillary and post-trial care | 30 | Provisions, if any, for ancillary and post-trial care, and for compensation to those who suffer harm from trial participation | ______________ |
| Dissemination policy | 31a | Plans for investigators and sponsor to communicate trial results to participants, healthcare professionals, the public, and other relevant groups (eg, via publication, reporting in results databases, or other data sharing arrangements), including any publication restrictions | ______14______ |
| | 31b | Authorship eligibility guidelines and any intended use of professional writers | ______________ |
| | 31c | Plans, if any, for granting public access to the full protocol, participant-level dataset, and statistical code | ______________ |
| **Appendices** | | | |
| Informed consent materials | 32 | Model consent form and other related documentation given to participants and authorised surrogates | ______18______ |
| Biological specimens | 33 | Plans for collection, laboratory evaluation, and storage of biological specimens for genetic or molecular analysis in the current trial and for future use in ancillary studies, if applicable | ______________ |

*It is strongly recommended that this checklist be read in conjunction with the SPIRIT 2013 Explanation & Elaboration for important clarification on the items. Amendments to the protocol should be tracked and dated. The SPIRIT checklist is copyrighted by the SPIRIT Group under the Creative Commons "Attribution-NonCommercial-NoDerivs 3.0 Unported" license.

For peer review only
